# DFT, Molecular Docking, and Molecular Dynamics Analysis of Electronic Structure, Reactivity, and PTP1B Binding of Phenylacetic and Benzylmalonic Acid-Functionalized C_60_ Derivatives

**DOI:** 10.3390/molecules31193543

**Published:** 2026-10-04

**Authors:** Verónica Rodríguez-Celestino, Zuriel Natanael Cisneros-García, Jaime Gustavo Rodríguez-Zavala, Carlos Iván Méndez-Barrientos, José Guadalupe Facio-Muñoz

**Affiliations:** Universidad de Guadalajara, Centro Universitario de los Lagos, Departamento de Ciencias Exactas y Tecnología, Av. Enrique Díaz de León 1144, Lagos de Moreno 47460, Jalisco, Mexico; veronica.rodriguez2941@alumnos.udg.mx (V.R.-C.); carlos.mendez8057@academicos.udg.mx (C.I.M.-B.); jose.facio@academicos.udg.mx (J.G.F.-M.)

**Keywords:** DFT calculations, molecular docking, molecular dynamics, water-soluble fullerenes, protein tyrosine phosphatase 1B (PTP1B)

## Abstract

Water-soluble C_60_ derivatives functionalized with phenylacetic acid (PhAA) and benzylmalonic acid (BnMA), together with their di- and tetrahydroxylated derivatives, were investigated using density functional theory, molecular docking, and molecular dynamics simulations to evaluate their electronic structure, local and global reactivity, and interactions with protein tyrosine phosphatase 1B (PTP1B). Successive hydroxylation steps were thermodynamically favorable, while electronic structure and reactivity analyses indicated that the fullerene cage retains features compatible with further ROS-related reactivity after hydroxylation. Electrostatic potential analysis revealed differentiated polarity between the fullerene and functionalization regions. Docking showed functionalization-dependent binding modes: PhAA-derived systems sampled both peripheral regions near the WPD and R loops and the catalytic pocket, whereas BnMA-derived systems consistently occupied the catalytic site and interacted with key residues, including Cys215 and Arg221. Molecular dynamics showed reduced flexibility in functionally relevant regions, reorganization of correlated motions, and average catalytic-pocket conformations that were more open than in apo-PTP1B. Overall, these results support the potential of these derivatives for ROS-related reactivity and modulation of PTP1B, although experimental validation is required to establish their biological activity.

## 1. Introduction

Protein tyrosine phosphatase 1B (PTP1B) is an intracellular phosphatase encoded by the *PTPN1* gene that acts as a negative regulator of signaling pathways mediated by receptor tyrosine kinases and cytosolic kinases [1]. Structurally, PTP1B consists of 435 amino acids and is considered the prototype of class I protein tyrosine phosphatases. Its catalytic domain comprises residues 1–300 and contains several structural motifs involved in the regulation of enzymatic activity. These include the P-loop (His214–Arg221), which contains the key catalytic residues Cys215 and Arg221; the WPD-loop (Thr177–Pro185), which contains Asp181, an essential residue involved in active-site closure; the Q-loop (Ile261–Asp265) and R-loop (Val113–Ser118), which contribute to substrate positioning and anchoring; the S-loop (Ser201–Gly209); and the α3 (Glu186–Glu200), α6 (Ala264–Ile281), and α7 (Val287–Asp298) helices, which contribute to structural stability, conformational flexibility, and maintenance of the active-site architecture. In addition, residues 301–435 form a disordered and highly hydrophobic C-terminal region that anchors the protein to the endoplasmic reticulum membrane [1,2,3,4,5,6,7].

PTP1B has attracted considerable attention because of its role in energy metabolism and several disease-associated signaling pathways. It negatively regulates insulin and leptin signaling by dephosphorylating key components of both pathways. In the insulin pathway, PTP1B primarily dephosphorylates the insulin receptor and insulin receptor substrates, thereby attenuating PI3K/AKT signaling and reducing cellular insulin sensitivity [8,9,10,11,12,13,14,15,16,17,18]. In the leptin axis, PTP1B attenuates JAK2/STAT3 signaling, resulting in reduced anorexigenic gene expression and increased orexigenic gene expression, thereby promoting appetite [6,13,14,19]. These regulatory effects directly link PTP1B to obesity and diabetes. In addition, impaired insulin and leptin signaling in the brain has been associated with neurological dysfunction. Insulin receptor activation in the hippocampus contributes to neuronal plasticity, learning, and memory, whereas leptin participates in neurogenesis, synaptogenesis, and the regulation of dendritic morphology [20,21,22]. PTP1B has also been implicated in the BDNF/TrkB pathway, where its overexpression alters TrkB phosphorylation, attenuates downstream signaling, and compromises BDNF/TrkB activity [23]. Therefore, PTP1B inhibition has been proposed as a promising strategy for ameliorating metabolic and cognitive dysfunction, including alterations associated with Alzheimer’s disease [24,25,26].

Beyond metabolic and neurological disorders, PTP1B has also been implicated in other pathological processes. It is overexpressed in several breast and ovarian cancers, particularly in tumors with HER2/Neu overexpression [1,6,27]. Genetic knockout studies in PTP1B^−/−^ mice have shown that tumorigenesis is markedly reduced and, in some cases, prevented [28]. In addition, Mo et al. reported that targeting PTP1B with roburic acid attenuated glucocorticoid-induced osteonecrosis of the femoral head [29].

Despite its therapeutic relevance, PTP1B remains a challenging drug target. Two main features complicate the development of effective inhibitors. First, the active site is highly conserved among PTP family members, which limits the selectivity of competitive inhibitors and increases the risk of affecting other phosphatases and their associated signaling pathways. Second, the catalytic pocket has a strongly positive electrostatic character, favoring the binding of negatively charged substrate-mimicking ligands that often exhibit poor membrane permeability [13,14,19].

To overcome these limitations, several alternative strategies have been explored. Allosteric inhibition may improve selectivity by targeting less conserved regions of the protein. Wiesmann et al. identified a nonconserved pocket located approximately 20 Å from Cys215, between the α3 and α6 helices, as a promising allosteric site [30]. In addition, Haider et al. highlighted secondary sites at the periphery of the catalytic pocket that are less conserved across the PTP family [14]. Bidentate inhibitors represent another alternative because they can simultaneously interact with the active site and a peripheral nonconserved site [1,6,13,14,19]. More recently, proteolysis-targeting chimera(PROTAC) degraders have emerged as a different approach. These heterobifunctional molecules consist of a ligand that binds the target protein, such as PTP1B, and a second ligand that recruits an E3 ubiquitin ligase, with both ligands connected by a linker. The resulting ternary complex promotes ubiquitination of the target protein and its subsequent recognition and degradation by the proteasome [1,13,31,32].

Within the search for alternative molecular scaffolds capable of interacting with PTP1B, water-soluble fullerene derivatives (WSFDs) represent attractive systems because they combine reported biocompatibility, potential therapeutic activity, and reactive oxygen species (ROS)-scavenging properties. Experimental, theoretical, and combined studies have reported both the antiradical activity of WSFDs and their ability to interact with molecular targets of therapeutic interest [3,4,5,31,33,34,35,36,37,38,39,40,41,42,43,44,45,46,47]. Among these systems, carboxylic acid-functionalized fullerene derivatives are particularly interesting because their functional groups may increase polarity and favor interactions with biological environments, whereas the fullerene cage may retain hydrophobic and ROS-scavenging characteristics [48].

In the present work, two carboxylic acid-functionalized WSFDs are evaluated: one bearing five phenylacetic acid-derived aryl substituents, –*p*–C_6_H_4_CH_2_COOH, and the other bearing five benzylmalonic acid-derived aryl substituents, –*p*–C_6_H_4_CH_2_CH(COOH)_2_. Here, *p* indicates that the carboxyl-containing side chain occupies the *para* position relative to the bond connecting the phenyl ring to the fullerene cage. For clarity, the derivatives containing phenylacetic acid- and benzylmalonic acid-derived substituents are hereafter abbreviated as PhAA and BnMA respectively. Their two- and three-dimensional structures are shown in Figure 1. Both compounds, originally obtained through halogen-substitution reactions, retain one chlorine atom covalently bound to the fullerene cage [48]. They exhibit a well-defined functionalization pattern in which all aryl carboxylic acid substituents are attached to the same region of the fullerene cage, whereas the opposite region remains intact. This asymmetric arrangement gives the molecules an amphiphilic character, with a polar functionalized region and a hydrophobic fullerene region, which may favor their interaction with protein targets.

Experimentally, these compounds have shown significant anti-HIV activity and low toxicity. Troshina et al. reported that both derivatives bind to the active site of HIV protease through the fullerene region, suggesting that the intact portion of the fullerene cage can participate directly in protein recognition [48]. Furthermore, because a substantial portion of the fullerene cage remains unmodified, these derivatives may retain relevant ROS-scavenging properties. Accordingly, the present work provides a multiscale computational investigation of these systems, ranging from the characterization of their electronic and antiradical properties to the simulation of their interactions with PTP1B.

## 2. Computational Details

All electronic structure calculations, including geometry optimizations, vibrational-frequency analyses, Fukui-function isosurfaces, and single-point calculations of vertical ionization energies and electron affinities, were performed at the B3LYP/6-311G(d,p) level of theory [49,50,51,52,53], as implemented in Gaussian 16 [54]. The B3LYP functional provides an adequate description of the electronic structure of fullerene-based systems, whereas the triple-ζ 6-311G(d,p) basis set provides sufficient flexibility to describe both the π-conjugated fullerene cage and the local rehybridization induced by functionalization. Thus, this level of theory represents a practical balance between computational cost and the description of structural, vibrational, and electronic properties. In addition, atom-averaged electrostatic potential (ESP) values were calculated using the post-processing program Multiwfn [55,56].

Prior to the docking simulations, the crystallographic structure of PTP1B (PDB ID: 1NL9) was prepared for the calculations [57]. Missing residues were modeled using CHARMM-GUI [58], and the resulting protein structure was energy-minimized in GROMACS using the steepest-descent algorithm and the GROMOS 54A7 force field [59,60]. Minimization was performed until the maximum force was below 1000 kJ mol^−1^ nm^−1^. Long-range electrostatic interactions were treated using the particle mesh Ewald (PME) method, whereas short-range electrostatic and van der Waals interactions were evaluated using a cutoff of 1.0 nm. Periodic boundary conditions were applied in all directions.

The minimized system was subsequently equilibrated under the NVT and NPT ensembles for 1 ns each using a 2 fs integration step and position restraints on the protein. Bonds involving hydrogen atoms were constrained using the LINCS algorithm. During both equilibration stages, the temperature was maintained at 300 K using the velocity-rescaling thermostat with separate coupling groups for the protein and the remaining components of the system. During NPT equilibration, the pressure was maintained at 1 bar using the Parrinello–Rahman barostat with isotropic coupling. Long-range electrostatic interactions were treated using PME, while short-range electrostatic and van der Waals cutoffs were set to 1.0 nm.

The ligand geometries used for molecular docking corresponded to local minima obtained by DFT optimization at the B3LYP/6-311G(d,p) level of theory. All carboxylic acid groups were modeled in their protonated form (–COOH), resulting in a net charge of zero for all ligands. A single protonation state was adopted consistently for the complete series to allow the effects associated with molecular functionalization and successive hydroxylation to be compared without introducing additional differences arising from changes in ligand ionization. As an additional structural reference, this choice is consistent with the neutral representation of the co-crystallized ligand 989 in the 1NL9 structure, which contains two protonated carboxylic acid groups [57]. Molecular docking simulations were performed using AutoDock Vina 1.1.2 [61]. Five independent runs were carried out for each ligand, and 20 binding poses were retained from each run, yielding a total of 100 poses per ligand. The search grid was centered on the active site of PTP1B and had dimensions of 55.33×51.25×51.14 Å along the *x*, *y*, and *z* axes, respectively, encompassing the catalytic pocket and the main structural motifs involved in PTP1B catalytic function, including the recognition, WPD, P, R, and Q loops, the secondary binding site and the α3 and α6 helices. The exhaustiveness parameter was set to 100, the maximum number of binding modes was set to 20, and the energy range was set to 3 kcal mol^−1^. Redocking of the co-crystallized inhibitor 989 was performed to evaluate the docking protocol [62,63,64], and the corresponding results are provided in the Supporting Information.

All docking poses generated by AutoDock Vina were subjected to hierarchical clustering using an RMSD cutoff of 8 Å. Although this cutoff is relatively large, the studied ligands contain a highly symmetric fullerene cage, and poses located within the same protein region can exhibit large RMSD values because of differences in their molecular orientation. Therefore, the selected cutoff allowed poses associated with different binding regions to be separated while grouping alternative orientations within the same region. For each ligand, the representative binding pose was selected as the lowest-energy structure within the most populated cluster. Protein–ligand interactions in the selected representative poses were analyzed using LigPlot+ 2.2.9 [65] and the open-source version of the PyMOL Molecular Graphics System, version 2.5 [66].

Molecular dynamics simulations were performed for apo-PTP1B and the selected protein–ligand complexes to evaluate their structural stability and conformational evolution. To maintain consistency between the protein and ligand parameterizations, ligand topologies were generated using the Automated Topology Builder (ATB) [67], with parameters compatible with the GROMOS 54A7 force field. Each system was solvated with the SPC water model in a dodecahedral box with a minimum solute–box distance of 1.0 nm. Na^+^ or Cl^−^ counterions were added as required to neutralize the systems, without imposing an additional salt concentration. The solvated and neutralized systems were then energy-minimized using the steepest-descent algorithm until the maximum force was below 1000 kJ mol^−1^ nm^−1^. Equilibration consisted of 1 ns under the NVT ensemble at 300 K followed by 1 ns under the NPT ensemble at 300 K and 1 bar, with positional restraints applied to the ligand during both stages. Temperature was controlled using the velocity-rescaling thermostat, while pressure during the NPT stage was controlled using the Parrinello–Rahman barostat. Production consisted of a single 100 ns trajectory for each system at 300 K and 1 bar, using the same thermostat and barostat and without positional restraints. Long-range electrostatic interactions were treated using the particle mesh Ewald (PME) method, whereas short-range electrostatic and van der Waals interactions were evaluated using a cutoff of 1.0 nm. Periodic boundary conditions were applied in all directions. Root-mean-square deviation (RMSD), root-mean-square fluctuation (RMSF), and principal component analysis (PCA) were calculated using tools implemented in GROMACS, whereas dynamic cross-correlation matrices (DCCMs) were obtained using the MDAnalysis 2.10.0 library [68,69].

## 3. Results and Discussions

### 3.1. Electronic Structure

#### 3.1.1. Determination of the Minimum-Energy Structures of Hydroxylated Derivatives

The molecular geometries of PhAA and BnMA reported by Troshina et al. [48] were optimized at the B3LYP/6-311G(d,p) level of theory (Figure 1). Vibrational-frequency analyses were subsequently performed at the same level of theory, and the absence of imaginary frequencies confirmed that the optimized structures correspond to minima on the potential energy surface.

The radical-scavenging potential of PhAA and BnMA was estimated through a combined local-reactivity and thermodynamic analysis. To identify the fullerene-cage regions most susceptible to radical attack, the Fukui function $f^{0}\left(r\right)$ was calculated using the finite-difference approximation, as expressed in Equation (1) [70,71],

(1) $$f^{0}\left(r\right)≅\frac{\rho_{N+1}\left(r\right)-\rho_{N-1}\left(r\right)}{2}.$$

Figure 2 shows the fullerene-cage sites with the highest susceptibility toward radical attack. More than one carbon atom exhibits a nonzero contribution to the Fukui function. Therefore, the isosurface value was gradually increased to distinguish the most susceptible site, which was selected as the last carbon atom retaining a visible Fukui-function contribution. Once this position was identified, the addition of the second hydroxyl group was explored at nearby carbon atoms, considering sites connected through [6,6] and [5,6] bonds, as well as carbon atoms located in the *para* position relative to the initially selected site. All proposed structures were then optimized, and their relative energies were compared to identify the most favorable configuration. The thermodynamic favorability of the first hydroxylation step was evaluated using the overall reaction $\mathrm{WSFD}+\begin{array}{c} H_{2}O_{2} \end{array}\to \mathrm{WSFD}{\left(\mathrm{OH}\right)}_{2}$ as an idealized thermodynamic model to compare the separated reactants with the corresponding hydroxylated product. This overall reaction represents the initial and final states considered in the thermodynamic analysis and is not intended to describe the reaction mechanism. The reaction Gibbs free energy change (ΔG) was calculated as the difference between the Gibbs free energies of the products and reactants, $\Delta G=G_{\mathrm{products}}-G_{\mathrm{reactants}}$. The same procedure was subsequently applied to the second hydroxylation step, represented by the overall reaction $\mathrm{WSFD}{\left(\mathrm{OH}\right)}_{2}+\begin{array}{c} H_{2}O_{2} \end{array}\to \mathrm{WSFD}{\left(\mathrm{OH}\right)}_{4}$. The complete energetic comparison of the evaluated structures is provided in Table S1 of the Supplementary Materials.

##### Hydroxylation of PhAA

For PhAA, the Fukui-function analysis identifies C25 as the most susceptible site for radical attack (Figure 2a). Among the structures proposed for the first hydroxylation step, the most favorable configuration corresponds to hydroxylation across a [6,6] bond, with an associated ΔG value of −37.07 kcal/mol. As shown in Table 1, this hydroxylation is accompanied by a decrease in the dipole moment from 7.44 D for PhAA to 6.43 D for PhAA-2OH. For the second hydroxylation step the Fukui-function identifies C20 as the most susceptible site (Figure 2c), the most favorable structure also results from hydroxylation across a [6,6] bond, with a ΔG value of −43.45 kcal/mol, indicating that this process is also thermodynamically favorable. The dipole moment decreases further to 4.25 D for PhAA-4OH. Thus, successive hydroxylation steps of PhAA are thermodynamically favorable and are accompanied by a progressive decrease in the molecular dipole moment, suggesting that hydroxylation modifies the charge distribution within the fullerene derivative.

Beyond the thermodynamic and dipole-moment changes associated with hydroxylation, successive hydroxylation also affects the frontier electronic structure of PhAA. Figure 3 shows the HOMO and LUMO isosurfaces together with the corresponding HOMO–LUMO energy gaps ($\Delta_{HL}$). The first hydroxylation step produces only a small change in $\Delta_{HL}$, with a difference of 0.04 eV between PhAA and PhAA-2OH. In contrast, after the second hydroxylation step, PhAA-4OH exhibits a more pronounced reduction in $\Delta_{HL}$, approximately 0.3 eV lower than those of PhAA and PhAA-2OH. Inspection of the orbital isosurfaces also reveals noticeable differences in their spatial distribution. Such changes may influence the electronic reactivity of the derivatives and the molecular regions involved in charge-transfer processes. For PhAA (Figure 3a), the HOMO is mainly distributed over the conjugated π system of the fullerene cage, whereas the LUMO is also predominantly delocalized over the fullerene moiety, with a spatial distribution consistent with a mainly $\pi^{*}$-like character. In PhAA-2OH (Figure 3b), the HOMO shows a more heterogeneous distribution, with contributions from the conjugated π system of the fullerene cage and from the oxygen atoms of the hydroxyl groups. The LUMO remains broadly delocalized over the fullerene cage, with only minor contributions from the hydroxylated region, indicating that the first hydroxylation step does not substantially alter its fullerene-centered character. For PhAA-4OH (Figure 3c), the HOMO distribution is again similar to that observed for PhAA, although a small contribution from one of the hydroxyl oxygen atoms is also present. The LUMO remains mainly distributed over the fullerene cage, with additional contributions from the functionalized regions. Overall, successive hydroxylation steps preserve the predominantly fullerene-centered character of the FMOs while producing local changes in their spatial distribution. These changes are particularly noticeable for PhAA-2OH, whereas PhAA-4OH exhibits a HOMO distribution more similar to that of PhAA. In contrast, the largest change in $\Delta_{HL}$ occurs after the second hydroxylation step.

##### Hydroxylation of BnMA

For BnMA, analysis of the Fukui function identifies C24 as the most susceptible atom for radical attack (Figure 2b). Similar to PhAA, the most favorable configuration for the first hydroxylation step involves hydroxylation across a [6,6] bond, with a ΔG value of −37.18 kcal/mol. For the second hydroxylation step, the most favorable configuration also involves hydroxylation across a [6,6] bond, formed by C37 and C36 (Figure 2d), yielding a ΔG value of −42.04 kcal/mol. The negative ΔG values indicate that both hydroxylation steps are thermodynamically favorable. In contrast to the behavior observed for the PhAA-derived systems, the dipole moment increases progressively from 4.52 D for BnMA to 4.65 and 5.45 D for BnMA-2OH and BnMA-4OH, respectively. This trend indicates that successive hydroxylation steps increase the molecular polarity of the BnMA-derived systems, which may modify their interaction with polar regions of biomolecular targets. In addition, the incorporated hydroxyl groups provide additional hydrogen-bond donors and acceptors. Together, these changes suggest that hydroxylation may alter the interactions of these derivatives with biological targets.

The frontier molecular orbital analysis of the BnMA-derived systems shows a behavior similar to that observed for the PhAA series. Figure 4a–c shows the spatial distributions of the HOMO and LUMO together with the corresponding $\Delta_{HL}$ values. Comparison with Figure 3 reveals that $\Delta_{HL}$ remains nearly unchanged after the first hydroxylation step, whereas the second hydroxylation step leads to a decrease of approximately 0.3 eV for BnMA-4OH. Similarities are also observed in the spatial distribution of the FMOs. For BnMA (Figure 4a), the HOMO is mainly distributed over the conjugated π system of the fullerene cage, whereas the LUMO is broadly delocalized over the fullerene moiety and exhibits a distribution consistent with a predominantly $\pi^{*}$-like character. After the first hydroxylation step, BnMA-2OH exhibits a local redistribution of the HOMO similar to that observed for PhAA-2OH, which may reflect contributions from both the conjugated π system of the fullerene cage and the oxygen atoms of the hydroxyl groups. In contrast, the LUMO remains predominantly fullerene-centered and broadly distributed over the cage. For BnMA-4OH, the HOMO distribution again resembles that of the non-hydroxylated BnMA system, although contributions from the hydroxyl oxygen atoms are also appreciable. Similarly, the LUMO remains mainly distributed over the fullerene cage, showing only local changes associated with functionalization. Overall, the BnMA-derived systems reproduce the main features observed for the PhAA series: the FMOs remain predominantly fullerene-centered, while the incorporation of hydroxyl groups produces local changes in their spatial distribution and the second hydroxylation step is accompanied by a more pronounced reduction in $\Delta_{HL}$.

The results obtained for the frontier molecular orbitals indicate that the frontier electronic structure of both PhAA- and BnMA-derived systems is mainly governed by the fullerene cage. Both the HOMO and LUMO remain predominantly distributed over this moiety, whereas the hydroxyl groups induce only local changes in their spatial distributions. A common trend is also observed for both families: the first hydroxylation step produces only a minor change in $\Delta_{HL}$, whereas the second hydroxylation step leads to a more pronounced decrease of approximately 0.3 eV. Interestingly, this energetic variation does not necessarily parallel the visual changes in the orbital distributions, since the tetrahydroxylated derivatives show FMO patterns that are, in several respects, similar to those of the corresponding non-hydroxylated systems. Importantly, successive hydroxylation steps do not eliminate the fullerene-centered electronic features of the derivatives, suggesting that hydroxylation does not suppress the electronic characteristics of the fullerene cage that are relevant to further radical attack. This observation is complemented by the thermodynamic favorability found for the second hydroxylation step. Overall, successive hydroxylation steps modify the frontier orbital energies and introduce local changes in the electronic distribution without substantially altering the dominant role of the fullerene cage.

#### 3.1.2. Predicted Antiradical Potential

The previous section showed that successive hydroxylation steps of PhAA and BnMA are thermodynamically favorable. However, hydroxylation may also modify their electronic properties and, consequently, their predicted propensity to participate in subsequent radical-scavenging processes. To examine this behavior, the present section analyzes how the global electronic reactivity descriptors evolve after successive hydroxylation steps. The vertical *I* and *A* values were calculated according to Equations (2) and (3), respectively [72,73].

(2) $$I=E^{+}\left(q_{0}\right)-E\left(q_{0}\right)$$

(3) $$A=E\left(q_{0}\right)-E^{-}\left(q_{0}\right),$$

where $E(q_{0})$ is the energy of the neutral system at its optimized molecular geometry, whereas $E^{+}\left(q_{0}\right)$ and $E^{-}\left(q_{0}\right)$ are the energies of the corresponding cationic and anionic systems, respectively, evaluated at the geometry of the neutral molecule. In general, a lower *I* value indicates a greater tendency to donate an electron, whereas a higher *A* value indicates a greater tendency to accept an electron. According to Table 1, the first hydroxylation step decreases *I* from 6.96 to 6.81 eV for PhAA and from 7.00 to 6.90 eV for BnMA, indicating an enhancement of the electron-donor character in both systems. After the second hydroxylation step, however, some differences emerge between the two families. The *I* value remains practically unchanged between PhAA-2OH and PhAA-4OH, with a difference of only 0.01 eV, whereas it decreases by 0.12 eV from BnMA-2OH to BnMA-4OH, indicating a further increase in the electron-donor character of the latter system. In contrast, the *A* values show a less uniform behavior. After the first hydroxylation step, only minor changes are observed for both derivatives. After the second hydroxylation step, however, *A* increases by 0.18 eV from PhAA-2OH to PhAA-4OH and by 0.11 eV from BnMA-2OH to BnMA-4OH.

In addition, to compare the relative electron-donor and electron-acceptor tendencies of the studied systems, Figure 5 shows a relative donor–acceptor map based on the general concept of the donor–acceptor map (DAM) [74,75]. In the present analysis, the ionization energy and electron affinity were normalized with respect to pristine C_60_ according to Equations (4) and (5),

(4) $$R_{I}=\frac{I_{L}}{I_{C_{60}}}$$

(5) $$R_{A}=\frac{A_{L}}{A_{C_{60}}}$$

where $I_{L}$ and $A_{L}$ are the vertical ionization energy and electron affinity of compound *L*, respectively, whereas $I_{C_{60}}$ and $A_{C_{60}}$ are the corresponding values calculated for pristine C_60_, also reported in Table 1. Pristine C_60_ was selected as the reference because it represents the parent framework shared by all the studied compounds and constitutes a well-established benchmark for electron acceptance and radical reactivity [76,77,78]. Therefore, this comparison allows the effects of functionalization and successive hydroxylation on the electron-transfer properties to be evaluated directly.

As shown in Figure 5, all derivatives exhibit $R_{I}<1$, indicating lower ionization energies and, consequently, a greater propensity to donate electrons than pristine C_60_. In parallel, their $R_{A}<1$ values indicate lower electron affinities and, therefore, a reduced propensity to accept electrons relative to C_60_. The simultaneous decrease in $R_{I}$ and $R_{A}$ indicates that functionalization and successive hydroxylation shift the electronic response of these systems from the predominantly electron-accepting character of pristine C_60_ toward a donor-favored behavior. This shift is electronically compatible with ROS-scavenging mechanisms involving electron donation. Together with the negative Gibbs free energies obtained for successive hydroxylation steps, these results support the thermodynamic and electronic feasibility of electron-transfer and radical-addition pathways, and therefore the predicted antiradical potential of the studied derivatives.

#### 3.1.3. Average Electrostatic Potential

The average electrostatic potential (ESP) is a valuable tool for analyzing how the functional groups and the degree of hydroxylation modify the electrostatic character of the studied molecules and how these systems might interact with target proteins. Table 2 shows the overall average ESP, calculated over all atoms, as well as the average ESP for the two distinguishable regions of each derivative: the fullerene region, comprising the cage carbons and the hydroxyl groups attached to them, and the functionalization region, comprising the atoms of the polycarboxylic groups. In addition, Figure S1 presents the atom-resolved ESP statistics, with each panel divided into the fullerene and functionalization regions.

According to the data in Table 2, the overall electrostatic character of PhAA, PhAA-2OH, and PhAA-4OH is negative; however, important differences can be identified among these systems. PhAA and PhAA-2OH exhibit overall average ESP values of −0.97 and −1.22 kcal/mol, respectively, whereas PhAA-4OH, although still negative, displays a value closer to neutrality (−0.06 kcal/mol). The fragment-based analysis shows that both regions of PhAA and PhAA-2OH present negative average ESP values, with the functionalization region providing the largest contribution to the overall negative character. This behavior may favor the orientation of the polycarboxylated region toward positively charged or polar residues. In contrast, PhAA-4OH presents average ESP values of opposite sign, with a slightly positive fullerene region and a negative functionalization region. This spatial differentiation may favor interactions with protein environments of complementary electrostatic character and influence the orientation adopted during binding.

Unlike the PhAA and its hydroxylated derivatives, BnMA and BnMA-2OH show slightly positive overall average ESP values (+0.20 and +0.12 kcal/mol, respectively), whereas BnMA-4OH yields a negative value of −0.24 kcal/mol. Nevertheless, the three BnMA systems exhibit a consistent fragment-specific pattern: the fullerene region presents a positive average ESP, while the functionalization region presents a negative value. Therefore, PhAA-4OH and the three BnMA systems display a spatial separation of electrostatic character between the two molecular regions. Such differentiation may facilitate directional interactions with protein regions of complementary charge or polarity and contribute to more defined binding orientations. In these systems, the positive ESP of the fullerene region may favor interactions with negatively charged environments, whereas the negative functionalization region may preferentially interact with positively charged or basic polar residues.

The ESP results reveal a spatially differentiated electrostatic profile that may favor molecular recognition by PTP1B: the predominantly negative functionalized side could promote complementarity with positively charged residues in the catalytic region and partially reproduce the anionic character of the phosphotyrosine-containing substrate. In addition, the well-established extended π-conjugated surface of the fullerene cage provides a plausible platform for nonpolar and π-mediated contacts with the protein. This electrostatic picture complements the other electronic structure results, including the donor-favored character of the derivatives relative to pristine C_60_ and the negative Gibbs free energies calculated for the formation of hydroxylated products, which support the electronic and thermodynamic feasibility of their radical-scavenging activity. Thus, at least within the scope of electronic structure-based simulations, the studied WSFDs exhibit properties supporting their potential for both PTP1B recognition and antioxidant activity. Nevertheless, these results provide an initial molecular-level indication; molecular docking is therefore required to examine their possible binding modes and specific interactions with PTP1B, followed by molecular dynamics simulations to evaluate the stability and evolution of the resulting complexes.

### 3.2. Molecular Docking

#### 3.2.1. PhAA

Following the clustering procedure described in Section 2, the representative interaction pose for each WSFD was selected as the pose with the most favorable docking score within the most populated cluster. This criterion combines the docking score of the pose with its recurrence among the generated configurations. Figure 6 provides an overview of the representative poses selected for each studied WSFD, whereas Figures S3 and S4 show the distribution of the docking poses among the clusters obtained using an RMSD cutoff of 8 Å.

For PhAA, the most populated cluster comprises approximately 50% of the identified poses and contains a pose with a docking score of −7.5 kcal/mol. For PhAA-2OH, the largest cluster encompasses about 35% of the docked poses and includes a pose with a docking score of −7.6 kcal/mol, which is comparable to that obtained for PhAA and is close to the most favorable score obtained for this system. In the case of PhAA-4OH, the cluster containing the pose with the most favorable docking score is also the most populated, accounting for approximately 40% of the identified poses and yielding a docking score of −8.10 kcal/mol. Overall, the docking scores obtained for PhAA and PhAA-2OH are comparable, whereas PhAA-4OH yielded a lower docking score.

Table 3 summarizes the non-covalent interactions identified using LigPlot+, whereas Figure 7 presents their three-dimensional representations. A first notable difference among the three systems is the binding region (Figure 6). Both PhAA and PhAA-2OH are docked at peripheral regions of the catalytic domain, consistent with a possible allosteric binding mode, whereas PhAA-4OH is located directly over the active site, suggesting a possible competitive binding mode. According to Table 3 and Figure 7, hydrophobic contacts constitute the predominant interactions for PhAA, PhAA-2OH, and PhAA-4OH. In particular, PhAA, which is docked above the WPD loop, establishes eight hydrophobic contacts and only two hydrogen bonds. Nevertheless, these hydrogen bonds involve Phe182, a residue belonging to the WPD loop, which is a structural motif involved in the closure of the catalytic domain. Several hydrophobic contacts are also established with residues within this region, particularly Thr178, Pro180, and Asp181, as well as with residues located near the WPD loop, including Glu186, Ser187, and Ser190. In addition, as shown in Figure 7a, the fullerene fragment of PhAA is primarily responsible for establishing hydrophobic contacts with Ser190, Tyr152, Ser187, Glu186, and Arg112, suggesting that this moiety acts as an anchoring region for the ligand.

For PhAA-2OH, the representative binding pose is located on one side of the R-loop. As summarized in Table 3, the interactions are also predominantly hydrophobic, with ten hydrophobic contacts and five hydrogen bonds identified. As shown in Figure 7b, several of these hydrophobic contacts involve the fullerene cage, particularly with Ile134, Phe135, Pro89, and Gly136. In contrast, the hydrogen bonds are mainly established between the carboxylic and hydroxyl groups of the ligand and protein residues such as Asp137. Regarding the structural motifs involved in the interaction, Val113 is particularly relevant because it belongs to the R-loop, a region involved in substrate anchoring. Additional interactions are established with residues in the adjacent region spanning from Ala122 to Glu136.

For PhAA-4OH, the representative binding pose is located in the active-site region, with the phenylacetic acid groups oriented toward the center of the catalytic pocket, in agreement with the electrostatic character identified through the ESP analysis. This orientation allows the polar functional groups to interact directly with residues within the catalytic pocket, whereas the fullerene cage primarily establishes hydrophobic contacts. According to Table 3 and Figure 7c, eight hydrophobic contacts and six hydrogen bonds were identified. Particularly relevant are the interactions with Cys215 and Arg221 of the P-loop. Cys215 acts as the catalytic nucleophile during the dephosphorylation reaction, whereas Arg221 contributes to substrate recognition and stabilization of the phosphate group within the active site. Therefore, simultaneous contacts with these residues may interfere with proper substrate accommodation and catalytic organization. Additional interactions involve the secondary binding site, the substrate-recognition loop, the WPD-loop, and the Q-loop/α6 helix region. Overall, this extended interaction pattern suggests that PhAA-4OH can establish interactions with several residues involved in substrate recognition and catalysis, providing a plausible structural basis for competitive binding.

#### 3.2.2. BnMA

In contrast to the PhAA-derived systems (PhAA, PhAA-2OH, and PhAA-4OH), the BnMA-derived systems show a more concentrated clustering of poses, with larger differences in the populations of the identified clusters. As shown in Figure S4, all docking poses of BnMA are distributed into only two clusters. The cluster associated with the most favorable docking score (−8.6 kcal/mol) contains only 21% of the identified poses, whereas the second cluster, associated with a score of −7.9 kcal/mol, comprises 79% of the poses, indicating that this configuration was sampled much more frequently. A similar behavior is observed for BnMA-2OH, for which the poses are also grouped into two clusters. The cluster associated with the most favorable docking score (−8.3 kcal/mol) represents 22% of the total poses, whereas the second cluster, with a score of −7.6 kcal/mol, contains 78% of them. For BnMA-4OH, the poses are distributed into three clusters associated with docking scores of −7.6, −7.1, and −6.8 kcal/mol, accounting for 86%, 10%, and 4% of the total poses, respectively. Figure 6d–f shows the binding regions identified for BnMA, BnMA-2OH, and BnMA-4OH, respectively. In all three cases, a clear interaction with the catalytic pocket of PTP1B is observed. This behavior may be related to the larger number of carboxylic acid groups in the benzylmalonic acid-derived substituents, which contain twice as many carboxylic acid groups as the phenylacetic acid-derived substituents, and may therefore influence the interaction specificity with PTP1B.

Analysis of the ligand–protein interactions identified in the docking simulations, summarized in Table 4 and illustrated in Figure 7d, shows that hydrogen bonds are the predominant interactions between BnMA and PTP1B, with fourteen hydrogen bonds compared with nine hydrophobic contacts. As shown in Table 4, BnMA interacts with several functionally relevant structural motifs, including the secondary binding site, the substrate-recognition site, and the WPD, Q, and R loops. In addition, BnMA establishes both polar and hydrophobic interactions with important residues of the catalytic site, including Cys215, Ser216, Ala217, Ile219, Gly220, and Arg221. The direct interaction with these residues supports a possible competitive binding mode.

Similarly, BnMA-2OH interacts with the same structural motifs identified for BnMA (Figure 7e). However, two fewer hydrogen bonds are observed, and the nature of some contacts changes after the first hydroxylation step. For example, the interaction with Cys215 changes from polar to hydrophobic, whereas the opposite behavior is observed for Ile219. Despite the reduction in the number of hydrogen bonds, the docking score changes by only 0.3 kcal/mol with respect to BnMA. This relatively small difference suggests that the first hydroxylation step does not indicate a substantial change in the predicted binding affinity of BnMA toward PTP1B.

After the second hydroxylation step, BnMA-4OH exhibits a docking score of −7.6 kcal/mol, indicating that the additional hydroxyl groups do not substantially reduce the predicted binding affinity toward PTP1B or prevent interactions with key residues such as Cys215 and Arg221 and other functionally relevant structural motifs. A similar interaction pattern is maintained within the catalytic domain, although the number of hydrogen bonds increases. As shown in Figure 7f, the binding orientation of BnMA-4OH is only slightly reoriented with respect to the previous systems, favoring the formation of additional hydrogen bonds between the carboxylic groups of the fullerene derivative and residues within the catalytic pocket. Although this increase in hydrogen bonds is not directly reflected in the docking score, the corresponding binding orientation is much more frequently sampled, since its cluster comprises more than 80% of the total docked poses (Figure S4).

Based on these results, the BnMA-derived systems preferentially occupy the catalytic pocket of PTP1B and interact directly with key P-loop residues, particularly Cys215 and Arg221. However, these interactions extend beyond the active site toward the secondary binding site, the substrate-recognition region, and the WPD, Q, and R loops, involving several structural elements related to substrate recognition and catalysis. The simultaneous interaction with these regions could favor ligand retention within the catalytic domain and interfere not only with substrate access and positioning but also with the conformational changes required for catalysis, particularly those involving the WPD and Q loops. The greater number of carboxylic groups may contribute to the more defined binding orientation and the more concentrated clustering pattern observed for these systems, whereas successive hydroxylation modifies the hydrogen-bonding pattern without substantially reducing the predicted affinity. Overall, these features support a plausible competitive binding mode for the BnMA-derived systems. Nevertheless, molecular dynamics simulations are necessary to determine whether these binding poses and interactions persist over time under explicit-solvent conditions and whether the ligands restrict loop mobility, induce conformational changes, or favor conformations that are less suitable for catalysis.

### 3.3. Molecular Dynamics

#### 3.3.1. Structural Stability and Complementary Interaction Analysis

To evaluate the behavior of the docked complexes under dynamic conditions, the molecular dynamics analysis was restricted to residues 1–281 to minimize the contribution of the highly flexible C-terminal portion of the simulated structure and focus on the evolution of the catalytic domain. Visualizations of the 100 ns molecular dynamics trajectories for the six protein–ligand complexes are provided as Videos S1–S6 in the Supplementary Materials. As a first analysis, the global structural stability of the protein–ligand complexes was evaluated through the root-mean-square deviation (RMSD) of the C_α_ atoms over the 100 ns simulations with respect to the corresponding initial structures. As shown in Figure 8a, during the initial part of the simulation all complexes exhibit higher RMSD values than apo-PTP1B, suggesting greater structural rearrangement after ligand binding. Nevertheless, the RMSD profiles of all complexes eventually reach relatively stable values and do not show a continuous increase over the simulation time. In contrast, the RMSD of apo-PTP1B begins to increase at approximately 60 ns, reaching values similar to those observed for the complexes, before stabilizing again near 80 ns. From approximately 60 ns until the end of the simulation, the PTP1B–PhAA-2OH complex exhibits the lowest RMSD values among the studied systems. Figure 8b additionally shows the most frequently sampled RMSD values and their corresponding relative frequencies. For all complexes, the predominant RMSD range accounts for more than 60% of the simulation, indicating that each system spends a substantial fraction of the trajectory around a preferred structural deviation. Particularly, the complexes with PhAA-2OH and PhAA-4OH show relative frequencies above 75% and 85%, respectively. Together with the favorable docking results discussed in Section 3.2, these observations indicate that the selected protein–ligand complexes remain structurally stable during the molecular dynamics simulations, with PhAA-2OH and PhAA-4OH showing the most persistent RMSD behavior among the studied complexes.

To examine the ligand-induced changes in protein flexibility in more detail, Figure 9 shows the root-mean-square fluctuation (RMSF) of the C_α_ atoms. In general, the RMSF values are reduced for the protein–ligand complexes relative to apo-PTP1B, indicating an overall decrease in protein mobility. This effect is particularly noticeable in structural motifs associated with the catalytic function of PTP1B, including the WPD-loop, which participates in catalytic closure; the S-loop, involved in substrate recognition; and the α3 helix, which contributes to structural stability. To a lesser extent, reduced fluctuations are also observed in the R-loop and the secondary binding site. In addition, Figures S5 and S6 show the B-factor mapped onto the protein backbone, where the color scale is referenced to apo-PTP1B and the backbone thickness represents the mobility within each system. These representations are consistent with the RMSF results and show a general reduction in backbone mobility in the presence of the ligands. The reduced flexibility observed in several regions involved in substrate recognition and catalytic motions may contribute to a decrease in PTP1B catalytic activity.

Changes in protein compactness and solvent exposure were evaluated through the radius of gyration ($R_{g}$) and solvent-accessible surface area (SASA), respectively. The SASA profiles (Figure S7) show an overall decrease during the simulations, followed by fluctuations within relatively similar ranges for apo-PTP1B and the PTP1B–ligand complexes. A comparable behavior is observed for $R_{g}$ (Figure S8). After the larger fluctuations observed during the initial part of the trajectories, the systems generally converge toward values of approximately 2.1–2.3 nm, indicating that only slight changes in the overall compactness of PTP1B occur during the final part of the simulations. Finally, protein–ligand hydrogen-bond analysis (Figure S9) shows a system-dependent behavior. The PhAA complex forms relatively few hydrogen bonds during most of the trajectory, whereas the PhAA-2OH and PhAA-4OH yield a larger number of contacts. The BnMA-derived systems generally show a higher number of hydrogen bonds, although substantial fluctuations are observed throughout the simulations. These results indicate that hydrogen-bond interactions are dynamic and depend on the ligand structure. Overall, the trajectories show relatively limited changes in the global compactness and solvent exposure of PTP1B, whereas the hydrogen-bonding patterns depend to a greater extent on the structure of the bound ligand.

#### 3.3.2. Dynamic Cross-Correlation Matrices Analysis

The dynamic cross-correlation matrices (DCCMs) were analyzed to evaluate the effect of ligand binding on the collective motions of PTP1B. In the matrices shown in Figure 10, correlated and anticorrelated motions are represented using a color scale ranging from light blue to orange. The apo system, shown in Figure 10a, displays an extended pattern of coupled motions among several structural motifs. In particular, the catalytic P-loop shows positive correlations with the WPD-loop, R-loop, and Q-loop, which are located close to each other in the three-dimensional structure, suggesting coordinated motions among motifs involved in substrate anchoring and catalysis. Positive correlations are also observed between the secondary binding site and regions close to the P-loop, as well as with the S-loop, Q-loop, and α6 helix, indicating coordinated motions with regions involved in substrate recognition and active-site stability.

As shown in Figure 10b, the interaction of PhAA with PTP1B produces a general reduction in the intensity of both positive and negative correlations relative to the apo-protein. In particular, the coordinated motions previously observed between the P-loop and the WPD-, S-, and R-loops are largely lost, whereas the positive correlation with the Q-loop is maintained, although with lower intensity. A similar reduction is observed for the secondary binding site, which retains a positive correlation with the Q-loop but to a lesser extent than in the apo system. The PhAA-2OH and PhAA-4OH complexes show a similar behavior (Figure 10c,d), with reduced positive correlation between the P- and Q-loops and an almost null correlation between the P-loop and the R- and WPD-loops. In addition, the correlated motion between the secondary binding site and the Q-loop is reduced, whereas that with the S-loop disappears. For PhAA-2OH, the positive correlation previously observed between the secondary binding site and the region following the catalytic site is also lost.

Figure 10 shows the DCCMs obtained for the BnMA-derived complexes. For BnMA (Figure 10e), the positive correlations between the P-loop and the R-, Q-, and WPD-loops are maintained, together with a more extended pattern of positively correlated motions from the central region of the sequence toward the C-terminal direction. At the same time, a substantial part of the anticorrelated regions observed in apo-PTP1B is attenuated or disappears, indicating an important reorganization of the overall correlation pattern. For BnMA-2OH (Figure 10f), both correlated and anticorrelated motions are generally attenuated relative to the apo system. However, the positive correlations between the P-loop and the R-, WPD-, and Q-loops are largely maintained, although with slightly lower intensity, whereas the positive correlation between the secondary binding site and the S-loop disappears. A similar behavior is observed for BnMA-4OH (Figure 10g) when compared with BnMA-2OH: the positive correlations of the P-loop with the R-, WPD-, and Q-loops are preserved, while the correlation between the secondary binding site and the S-loop observed in apo-PTP1B remains absent. In addition, the correlations involving the secondary binding site are generally attenuated, although this effect is less pronounced than for BnMA. Overall, the BnMA-derived systems preserve several of the coordinated motions involving the catalytic loops while producing more evident changes in the anticorrelated regions and in the correlations associated with the secondary binding site.

Considering the DCCM results as a whole, ligand binding produces important changes in the collective motions of PTP1B rather than only local variations in protein flexibility. The PhAA-derived systems show a marked attenuation or loss of several correlations involving the P-loop, WPD-loop, R-loop, Q-loop, and secondary binding site, indicating a reduction in the coordinated motions among structural motifs involved in substrate recognition and catalysis. In contrast, the BnMA-derived systems preserve several of the positive correlations among the catalytic loops, although substantial changes are observed in the anticorrelated regions and in the correlations involving the secondary binding site. These differences indicate that the two families modify the internal dynamics of PTP1B in distinct ways. Such perturbations may interfere with the coordinated motions required for substrate positioning and catalytic activity. In particular, for PhAA and PhAA-2OH, which were docked at peripheral regions of the catalytic domain, the loss of correlated motions involving the active-site loops is consistent with a possible allosteric effect. Overall, the DCCM analysis indicates that ligand binding modifies the coordinated motions among functionally relevant regions of PTP1B, providing an additional mechanism by which these derivatives may affect protein activity beyond their direct interactions with the catalytic pocket.

#### 3.3.3. Principal Component Analysis in a Common Conformational Space

To evaluate differences in the global conformational behavior of the catalytic domain, a common-space principal component analysis was performed. The trajectories of all systems were concatenated considering only the C_α_ atoms of residues 1–281, and a single covariance matrix was calculated. Each individual trajectory was then projected onto the same first two principal components. PC1 and PC2 accounted for 24.92% and 15.08% of the total variance, respectively, together representing 40% of the collective positional variance captured by the common PCA.

Figure 11 shows the projection of all trajectories onto the same eigenvectors; therefore, differences in the PC1–PC2 plane reflect differences in the conformational regions sampled by each system. The projected ensembles occupy distinguishable regions of the common PC1–PC2 space, although some degree of overlap is observed. The main differences can first be identified by comparing the PhAA- and BnMA-derived systems. As shown in Figure 11a–d, the PhAA, PhAA-2OH, and PhAA-4OH complexes remain closer to the conformational region sampled by apo-PTP1B, although their distributions are not identical. The centroid analysis in the common PC1–PC2 space indicates that PhAA-4OH and PhAA are the closest systems to apo-PTP1B, with centroid distances of 1.06 and 1.11 Å, respectively, whereas PhAA-2OH shows a larger displacement of 1.67 Å. In comparison, BnMA, BnMA-2OH, and BnMA-4OH display larger centroid displacements of 1.93, 4.75, and 3.75 Å, respectively. Interestingly, although BnMA, BnMA-2OH, and BnMA-4OH all interact with the active site of PTP1B, they occupy different regions of the common PC1–PC2 space. In addition to the centroid displacement, the dispersion of the projected points shows that each ligand differs in the extent of conformational sampling along PC1 and PC2. The PhAA-derived systems show substantial overlap with the apo region, although their dispersion is not uniform. Along PC1, PhAA and PhAA-2OH show slightly narrower ranges of 3.20 and 2.93 Å, respectively, compared with 3.42 Å for apo-PTP1B, whereas PhAA-4OH shows a broader range of 3.89 Å. A similar trend is observed along PC2, where PhAA and PhAA-2OH show ranges of 2.47 and 2.60 Å, respectively, compared with 2.73 Å for apo-PTP1B. The BnMA-derived systems show a less uniform behavior. Their PC1 ranges are broader than that of apo-PTP1B, whereas along PC2, BnMA and BnMA-2OH show narrower ranges and BnMA-4OH shows a slightly broader range than the apo system.

In contrast to the behavior discussed previously in Section 3.2, the common PCA suggests that interaction within the same binding region does not necessarily produce the same collective dynamical response. Instead, the response appears to depend on the binding orientation, the functionalization pattern, and the specific contacts established within or around the catalytic site. Thus, although the number of carboxylic acid and hydroxyl groups may favor interactions with the active site, their spatial arrangement likely contributes to the distinct collective conformational behavior observed for each ligand.

#### 3.3.4. Free-Energy Landscapes in the Common Principal Component Space

To further characterize the conformational redistribution observed in the common PCA, free-energy landscapes were constructed from the PC1–PC2 projections of each system. This analysis allowed the identification of the low-energy regions preferentially sampled by apo-PTP1B and the PTP1B–fullerene complexes within the same collective conformational space. The PCA-FEL map of apo-PTP1B, shown in Figure 12a, displays an extended free-energy landscape with several low-energy regions distributed throughout the PC1–PC2 space. Rather than being concentrated in a compact region, the landscape indicates that, in the absence of a ligand, PTP1B samples several regions of the common PCA space. In comparison, PhAA (Figure 12b) displays a landscape more concentrated around the origin, with the first three minima located close to each other, indicating a more localized organization of the low-energy regions than in the apo system. Conversely, PhAA-2OH (Figure 12c) shows a more fragmented landscape, with one low-energy region located toward negative values of PC1 and PC2 and another separated region toward higher values of both components. This distribution indicates that PhAA-2OH favors distinct low-energy regions within the common PC space. Similarly, PhAA-4OH also produces two distinguishable regions in the free-energy landscape: a lower-energy region containing the first three minima and another region toward lower values of PC1 and PC2, corresponding to slightly higher-energy conformational states.

As shown in Figure 12e, BnMA produces a broad low-energy region located toward positive values of PC1 and negative values of PC2, clearly occupying a different region from apo-PTP1B and the PhAA-, PhAA-2OH-, and PhAA-4OH-bound systems. Consistent with the behavior discussed in previous sections, although BnMA-2OH and BnMA-4OH interact with PTP1B in a region similar to that of BnMA, they do not exhibit the same dynamic evolution, and this difference is also reflected in their PCA-FEL maps. As shown in Figure 12f,g, BnMA-2OH shifts the low-energy region toward negative values of PC1 and positive values of PC2. Furthermore, its landscape is more elongated, with the first three minima distributed along a diagonal region, indicating that BnMA-2OH not only displaces the centroid of the conformational sampling but also favors a different low-energy region within the PC1–PC2 space. Similarly, BnMA-4OH generates an elongated landscape extending diagonally through positive values of PC1 and PC2; in contrast to BnMA, the low-energy region of BnMA-4OH is more localized toward higher values of PC1 and PC2, where the first three minima are located close to each other rather than being distributed along the entire diagonal region.

The PCA-FEL maps reveal that each fullerene derivative redistributes the low-energy regions of PTP1B in a system-dependent manner. Apo-PTP1B displays an extended landscape with several low-energy regions, whereas PhAA shows a more compact organization around a central region of the common PC1–PC2 space. The PhAA-2OH and PhAA-4OH systems produce more divided landscapes, indicating that changes in the functionalization pattern alter the organization of the projected conformational substates. This effect is more pronounced for the BnMA-derivatives. The BnMA, BnMA-2OH, and BnMA-4OH systems occupy distinct low-energy regions, with BnMA-2OH showing the largest displacement and a notably elongated landscape. Therefore, even when the ligands interact with the active site, their projected free-energy landscapes indicate distinct collective conformational responses. These results support the idea that active-site binding alone does not define the collective conformational response; rather, this behavior also depends on ligand orientation, functionalization pattern, and the specific protein–ligand contacts.

#### 3.3.5. Principal Component Analysis of Dominant Motions and Structural Changes

To identify the structural motifs involved in the dominant internal motions of PTP1B, as well as the average structural changes occurring during each molecular dynamics simulation, the displacement vectors associated with PC1 and PC2 were mapped onto the average structure of each system. As shown in Figure 13, the arrows represent the displacement from the average structure toward one extreme projection along PC1 and PC2. The first two principal components account for approximately 40% of the total C_α_ positional variance, providing a representation of the main collective motions sampled during the simulations. In Figure 13, the structural motifs are represented by different colors: purple for the WPD-loop, yellow for the P-loop, red for the R-loop, orange for the α3 helix, green for the α6 helix, magenta for the Q-loop, dark blue for the secondary binding site, and cyan for the recognition loop. In addition, Table 5 summarizes the RMSD between the initial and average structures of the C_α_ atoms of the catalytic domain (residues 1–281), together with the distance between the C_α_ atoms of Phe182 and Gly218, a commonly used structural indicator for the catalytic pocket of PTP1B [79].

For the apo system, the average structure shows an RMSD of 2.22 Å with respect to the initial structure, whereas the Phe182–Gly218 C_α_ distance decreases from 16.30 to 12.20 Å (Table 5). The displacement vectors associated with PC1 (Figure 13a) are distributed throughout the protein, with slightly larger magnitudes at the bottom of the structure according to the perspective shown in the figure, whereas the largest PC2 vectors are more evident in the upper region. Thus, the molecular dynamics simulation of apo-PTP1B suggests that the dominant internal motions are distributed throughout the structure, while the catalytic pocket tends to adopt a more closed configuration.

For the PhAA complex, the average structure shows a larger deviation from the initial structure, with an RMSD of 2.61 Å, while the catalytic-pocket distance increases considerably from 16.50 to 20.50 Å. As shown in Figure 13b, the largest displacement vectors associated with PC1 and PC2 are mainly located in the R-loop and WPD-loop, indicating that these motifs contribute strongly to the dominant internal motions. Therefore, in the presence of PhAA, the main conformational fluctuations are associated with reorganizations of these loops. These changes suggest that PhAA may produce important effects on the catalytic dynamics of PTP1B, consistent with a possible non-competitive inhibitory mechanism.

The PhAA-2OH complex shows the smallest deformation of the average structure, with an RMSD of 1.90 Å, while the catalytic-pocket distance decreases from 13.90 to 12.60 Å. The largest displacement vectors are mainly observed near the secondary binding site, at the bottom of the structure according to the perspective shown in Figure 13c. Although vectors associated with PC1 and PC2 are still present in the R- and WPD-loops, these regions no longer show the dominant internal motions. In addition, the PC1 and PC2 vectors in some structural regions point in opposite directions, suggesting a partial compensation of these motions that may contribute to the smaller structural deformation observed for this complex.

For PhAA-4OH, the average structure shows an intermediate deformation within the PhAA-derived series, with an RMSD of 1.97 Å, together with a slight opening of the catalytic pocket from 16.80 to 18.00 Å. Although this opening is smaller than that observed for PhAA, the average structure shown in Figure 13d exhibits a pronounced deformation of the WPD-loop, which is involved in catalytic closure. The figure also shows that PhAA-4OH reorganizes the dominant internal motions: the region close to the R-loop displays the most pronounced displacement vectors associated with PC1, whereas the secondary binding site and the surrounding residues show the largest vectors associated with PC2.

Regarding the BnMA-derived systems, BnMA induces an average structural deformation of 2.06 Å and a slight closure of the catalytic pocket from 16.30 to 14.04 Å. Interestingly, the displacement vectors associated with PC1 and PC2 (Figure 13e) are considerably reduced throughout the structure, indicating that BnMA significantly modifies the dominant internal motions of PTP1B and that the extreme projections along PC1 and PC2 remain relatively close to the average structure. This behavior is consistent with the DCCM analysis discussed in Section 3.3.2, where the overall correlation pattern of BnMA is markedly altered with respect to apo-PTP1B. Although positive correlations among several catalytic motifs are maintained, a substantial part of the anticorrelated motions observed in the apo system is attenuated or lost. These changes could indicate inhibitory activity by affecting the internal dynamics of PTP1B required for catalytic function.

The BnMA-2OH complex shows an average structural deformation of 1.99 Å, similar to that observed for BnMA. However, BnMA-2OH produces a greater opening of the catalytic pocket, with the Phe182–Gly218 distance increasing from 18.10 to 19.00 Å in the average structure. These values correspond to the second widest distances among the studied systems for both the initial and average structures. Although BnMA and BnMA-2OH interact with the same region of PTP1B, their particular binding orientations and specific interactions lead to clearly different dynamic evolutions, with BnMA-2OH favoring a more open catalytic-pocket configuration. As shown in Figure 13f, the displacement vectors associated with PC1 and PC2 are not reduced as observed for BnMA. PC1 vectors are evident in several structural motifs, including the WPD- and R-loops, the α3 and α6 helices, and regions near the secondary binding site and recognition loop. In contrast, the PC2 vectors are more uniformly distributed throughout PTP1B. Thus, although part of the internal motion is recovered in the presence of BnMA-2OH, the ligand also promotes a marked opening of the catalytic domain, which may correspond to a less active configuration.

Finally, BnMA-4OH induces the second largest deformation of the average structure, with an RMSD of 2.37 Å according to Table 5. In addition, the catalytic pocket undergoes a considerable closure, with the Phe182–Gly218 distance decreasing from 19.40 to 15.60 Å. As shown in Figure 13g, despite the common binding region shared with BnMA and BnMA-2OH, BnMA-4OH induces a different pattern of dominant internal motions. In this system, the largest displacement vectors associated with PC1 are mainly localized near the WPD- and R-loops, whereas those associated with PC2 are more pronounced at the bottom of the structure, according to the perspective shown in the figure.

The results of this section show that each ligand induces a distinct dynamic evolution of PTP1B, even when the binding region is similar. Importantly, despite these differences in binding mode and internal motions, all ligand-bound systems adopt average structures with a more open catalytic pocket than apo-PTP1B. This common tendency suggests that ligand binding shifts the catalytic domain toward configurations that may be less favorable for catalytic activity.

### 3.4. Limitations and Future Perspectives

The present study is based exclusively on computational approaches; therefore, the predicted interaction of the C_60_ derivatives with PTP1B, as well as their proposed antioxidant potential, requires experimental validation before their biological activity can be established. Experimental validation would first require sufficient quantities of the selected derivatives and recombinant PTP1B to perform enzymatic inhibition assays [4,5]. These assays could determine the half-maximal inhibitory concentration ($IC_{50}$), whereas a more detailed kinetic characterization would allow estimation of the inhibition constant ($K_{i}$) and elucidation of the inhibition mechanism. In parallel, the antioxidant potential of the functionalized fullerenes could be experimentally evaluated through H_2_O_2_- and radical-scavenging assays [39,40] intracellular oxidative damage, and cytocompatibility. Cell-based models, including those involving increased PTP1B expression and H_2_O_2_-induced oxidative stress, could then be used to assess PTP1B-dependent signaling, intracellular oxidative damage, and cytocompatibility through parameters such as the half-maximal cytotoxic concentration ($CC_{50}$) [39,40,80]. This integrated approach would help determine whether the compounds can combine enzymatic modulation with protection against oxidative stress. Finally, derivatives showing favorable enzymatic activity, antioxidant capacity, and cellular biocompatibility could be evaluated in appropriate murine models to assess their efficacy and safety in vivo [32].

## 4. Conclusions

A comprehensive computational study was conducted to investigate water-soluble C_60_ derivatives functionalized with phenylacetic acid- and benzylmalonic acid-derived groups, focusing on their electronic reactivity, radical-scavenging potential, and interaction with PTP1B. Electronic structure calculations showed that successive hydroxylation steps are thermodynamically favorable for both PhAA- and BnMA-derived systems. The vertical ionization energies and electron affinities indicate that these processes do not eliminate their ability to participate in subsequent electron-transfer events, while the frontier molecular orbital analysis shows that the fullerene cage remains the dominant region of the electronic structure after successive hydroxylation. The first addition produces only minor changes in the HOMO–LUMO gap, whereas the second decreases ΔHL by approximately 0.3 eV. The ESP analysis further shows differentiated electrostatic characteristics between the fullerene and functionalized regions, which may contribute to their orientation in protein environments of different polarity. Taken together, these results indicate that the initial capture of ROS does not suppress the characteristic electronic properties of the fullerene cage and support the possibility that the partially hydroxylated derivatives remain capable of participating in further radical-scavenging processes. These computational results describe the thermodynamic and electronic feasibility of the proposed radical-scavenging processes but do not establish their kinetics or experimental antioxidant capacity.

Molecular docking and molecular dynamics reveal that the biological implications of this progressive chemical modification are not limited to changes in predicted radical-scavenging behavior. The original and hydroxylated derivatives retain the ability to interact with PTP1B, but the preferred binding mode and the resulting protein dynamics depend strongly on the molecular system. The BnMA-derived systems show the most consistent tendency to occupy the catalytic pocket, with favorable docking scores, highly concentrated clustering in a few binding orientations, and direct interactions with catalytic residues such as Cys215 and Arg221, supporting their potential as competitive inhibitors. In contrast, the PhAA-derived series displays a broader range of interaction modes: PhAA and PhAA-2OH preferentially interact with peripheral regions associated with the WPD- and R-loop environments, whereas PhAA-4OH occupies the catalytic pocket and establishes direct interactions with catalytic residues. The molecular dynamics simulations further show that these different binding modes produce distinct effects on protein flexibility, correlated motions, conformational sampling, and free-energy landscapes. Importantly, even ligands occupying similar regions do not necessarily induce the same dynamic response, and all ligand-bound systems adopt average structures with a more open catalytic pocket than average apo-PTP1B.

From a molecular-design perspective, these results suggest that the PhAA-derived series may represent a particularly interesting platform for further investigation. Whereas the BnMA-derived systems provide more consistent evidence for direct competitive binding, the peripheral interaction of PhAA and PhAA-2OH offers the possibility of affecting catalytic dynamics without directly occupying the highly conserved active site, an aspect that may be advantageous when considering the selectivity challenges associated with PTP1B inhibition. At the same time, PhAA-4OH emerges as a complementary candidate within the same chemical family because it combines thermodynamically favorable hydroxylation with the direct interactions involving Cys215 and Arg221 and persistent effects on PTP1B dynamics. Therefore, rather than identifying a single optimal derivative, the present results point to the PhAAseries as a useful scaffold for exploring both peripheral/allosteric and competitive inhibition strategies. They also indicate that the original and hydroxylated forms should be considered separately in future studies, since ROS capture does not simply generate electronically inactive products but may produce derivatives that retain PTP1B-binding capability while adopting different interaction modes. This dual behavior provides a concrete basis for further evaluation of these systems as multifunctional compounds combining predicted radical-scavenging potential with potential modulation of PTP1B activity.

## Figures and Tables

**Figure 1:**
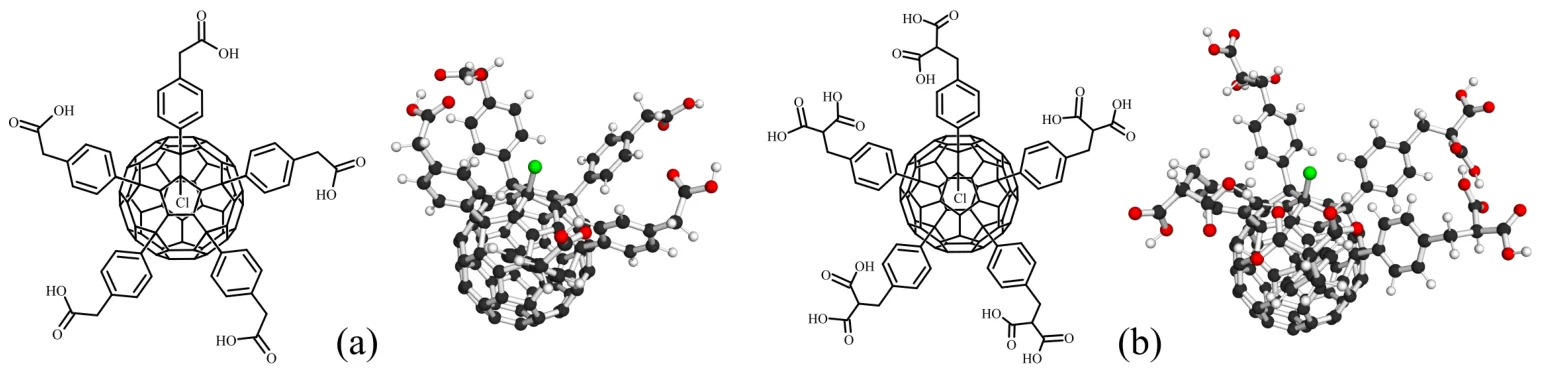
2D and 3D representations of the WSFD: (**a**) PhAA and (**b**) BnMA. In the 3D representations, carbon, oxygen, hydrogen, and chlorine atoms are shown in dark gray, red, light gray, and green, respectively.

**Figure 2:**
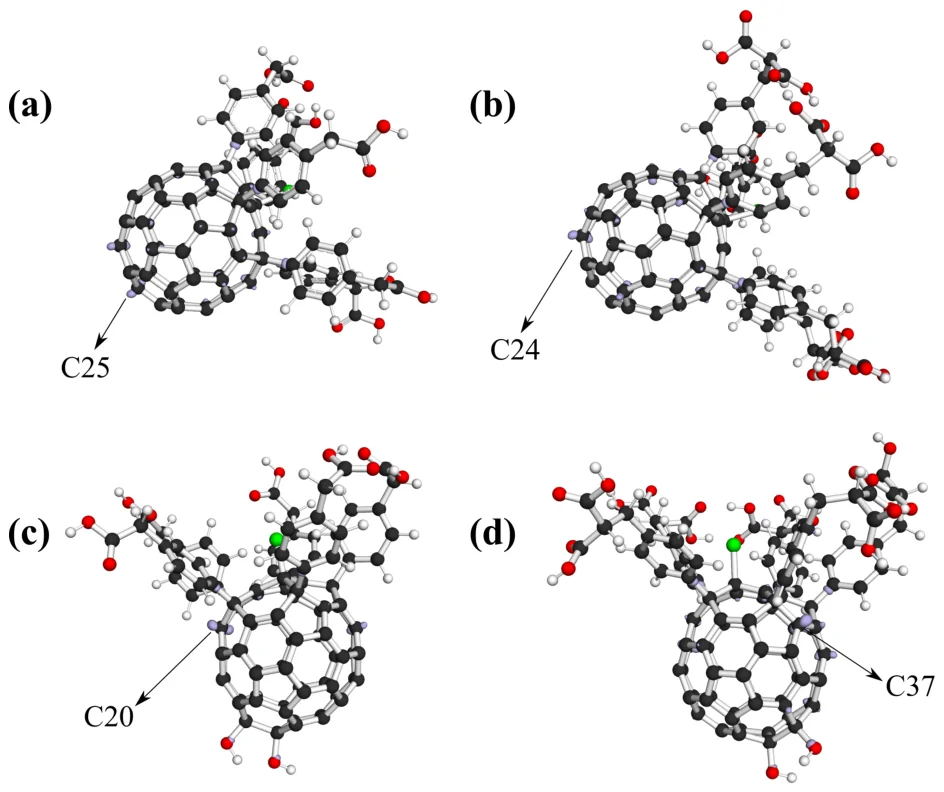
Fukui function $f^{0}\left(r\right)$ isosurfaces for (**a**) PhAA, (**b**) BnMA, (**c**) PhAA-2OH, and (**d**) BnMA-2OH. The Fukui-function isosurfaces are shown in light purple. The arrows indicate the carbon atoms identified as the most susceptible sites for radical attack: C25 and C24 for the original derivatives, and C20 and C37 after the first radical addition, respectively.

**Figure 3:**
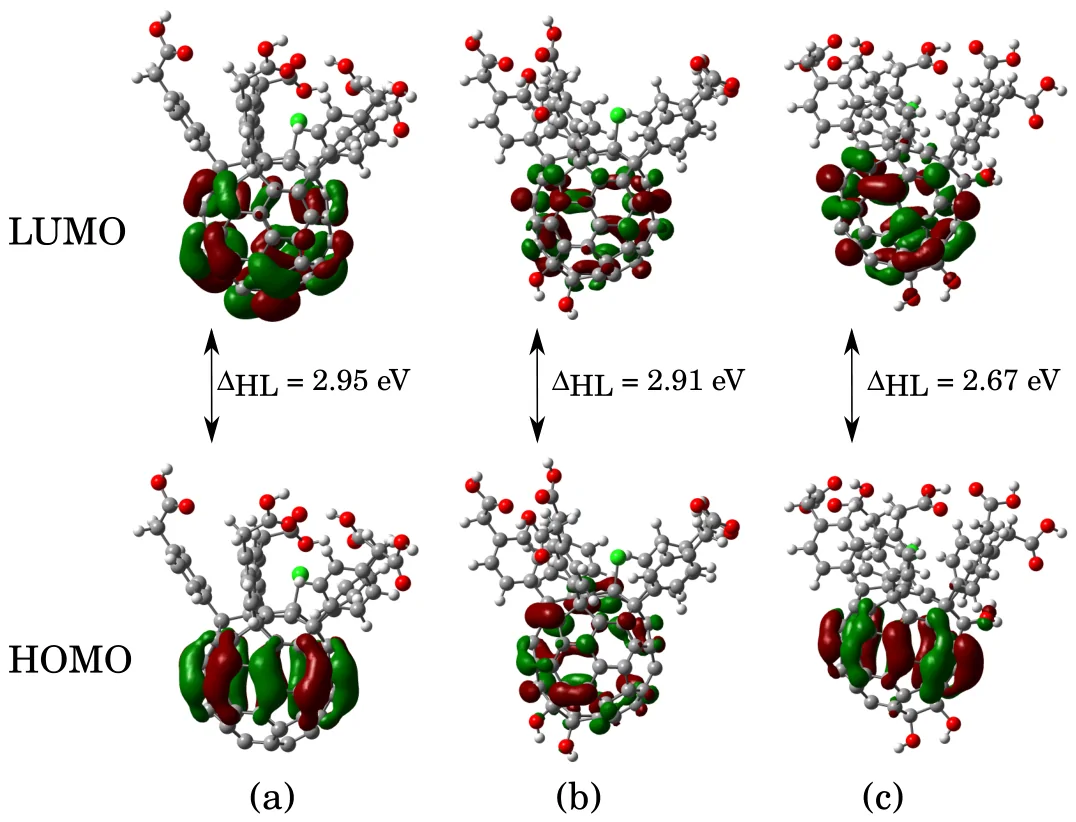
Frontier molecular orbital isosurfaces and corresponding HOMO–LUMO energy gaps ($\Delta_{HL}$) for (**a**) PhAA, (**b**) PhAA-2OH, and (**c**) PhAA-4OH. Green and red isosurfaces represent opposite phases of the molecular orbital wavefunctions.

**Figure 4:**
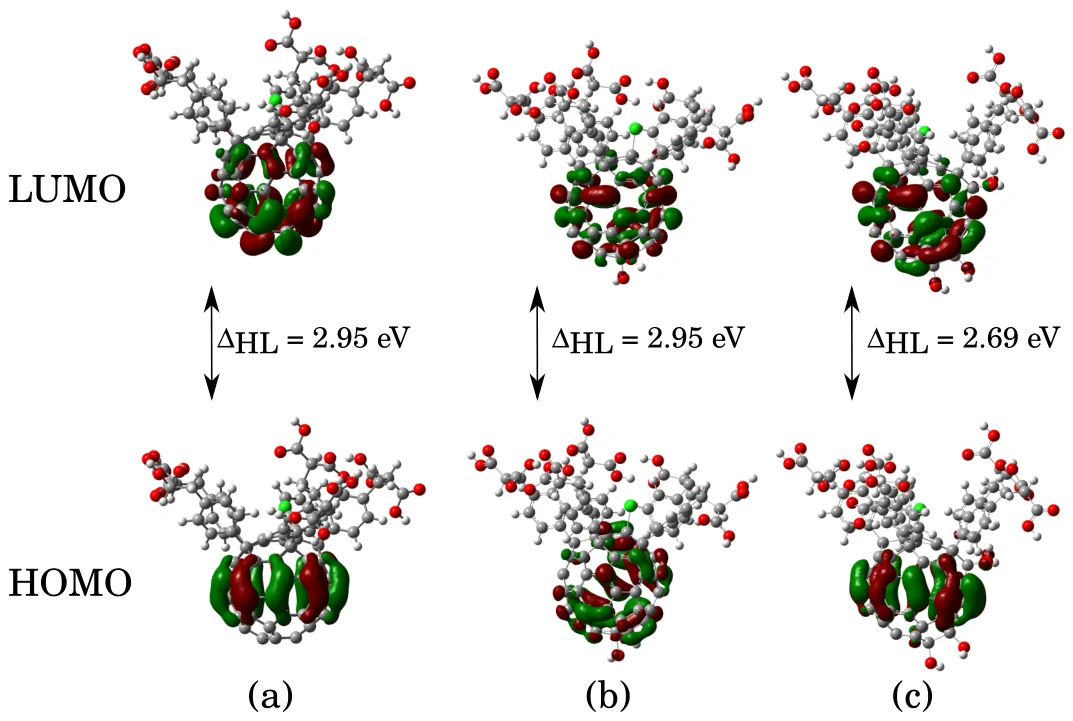
Frontier molecular orbital isosurfaces and corresponding HOMO–LUMO energy gaps ($\Delta_{HL}$) for (**a**) BnMA, (**b**) BnMA-2OH and (**c**) BnMA-4OH. Green and red isosurfaces represent opposite phases of the molecular orbital wavefunctions.

**Figure 5:**
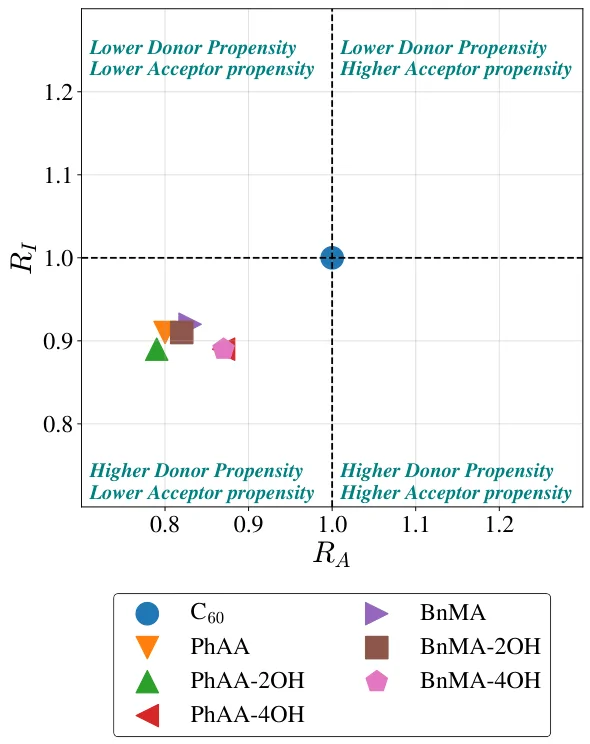
Full electron donor–acceptor map of the studied WSFDs relative to pristine C_60_, based on the normalized electron affinity ($R_{A}$) and ionization energy ($R_{I}$).

**Figure 6:**
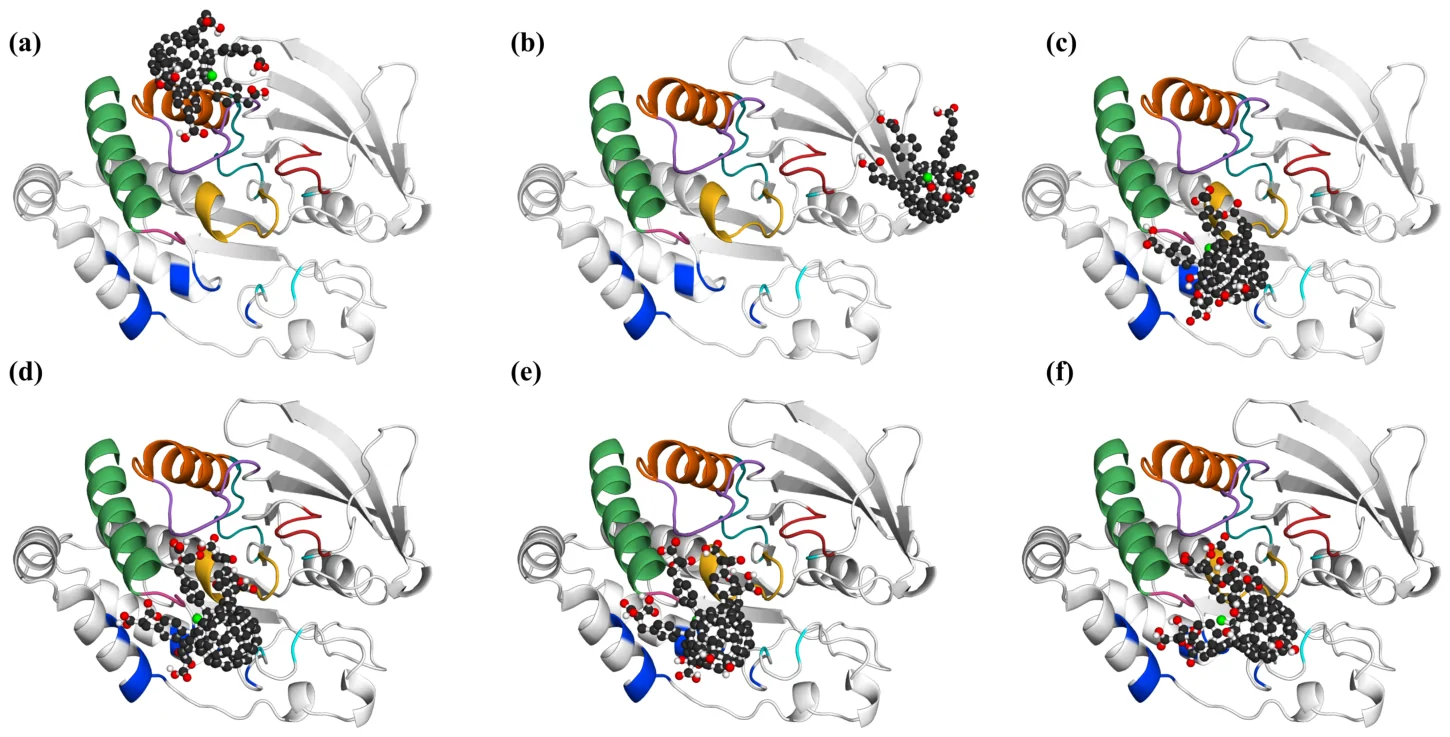
Representative docking poses of the studied WSFDs with PTP1B: (**a**) PhAA, (**b**) PhAA-2OH, (**c**) PhAA-4OH, (**d**) BnMA, (**e**) BnMA-2OH, and (**f**) BnMA-4OH. Relevant structural motifs of PTP1B are highlighted in different colors: purple for the WPD-loop, yellow for the P-loop, red for the R-loop, orange for the α3 helix, green for the α6 helix, magenta for the Q-loop, dark blue for the secondary binding site, and cyan for the substrate-recognition loop.

**Figure 7:**
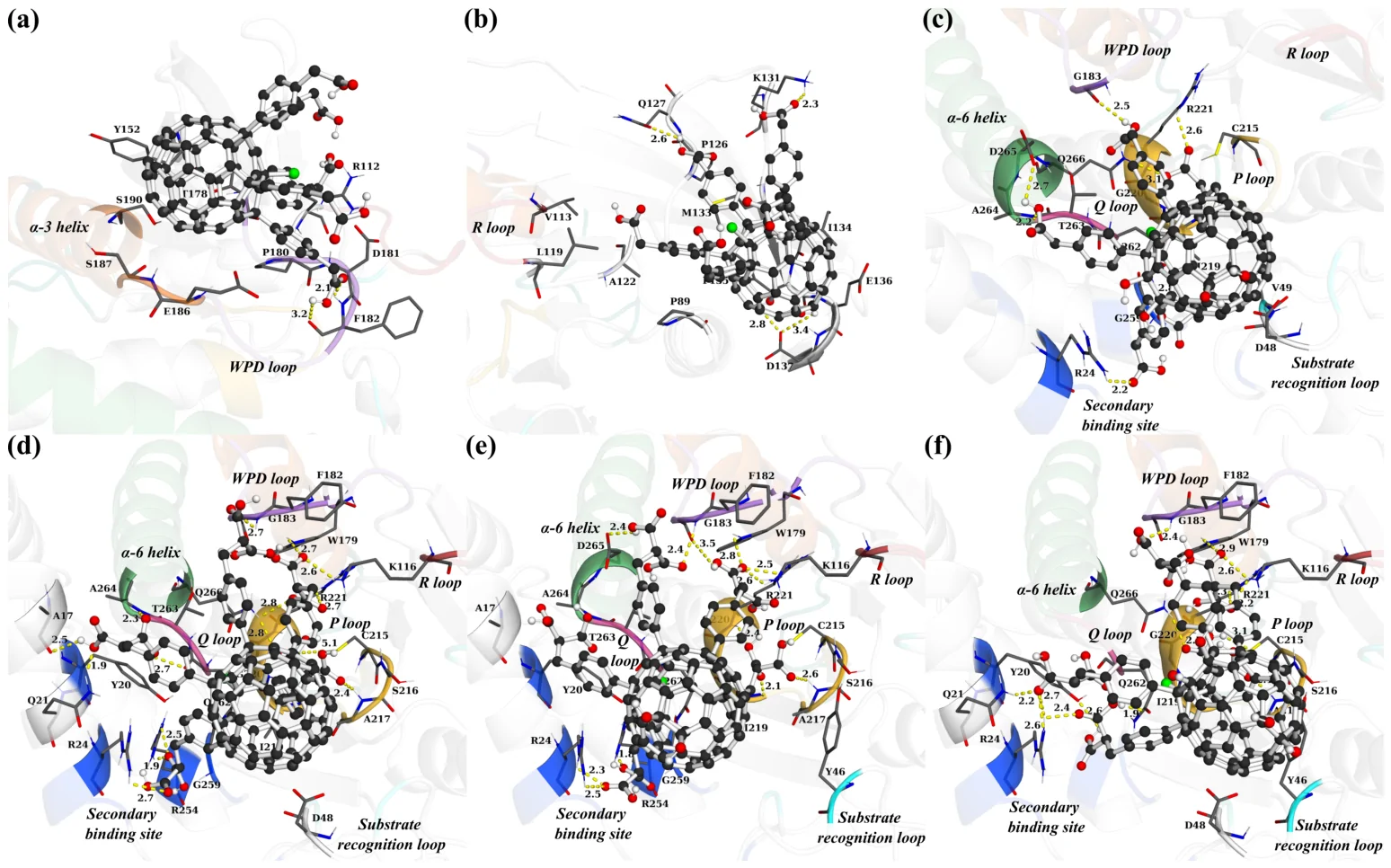
Detailed representation of the representative docking poses of (**a**) PhAA, (**b**) PhAA-2OH, (**c**) PhAA-4OH, (**d**) BnMA, (**e**) BnMA-2OH, and (**f**) BnMA-4OH with PTP1B, showing the main interacting residues and relevant structural motifs. For clarity, amino acid residues are labeled using the one-letter code.

**Figure 8:**
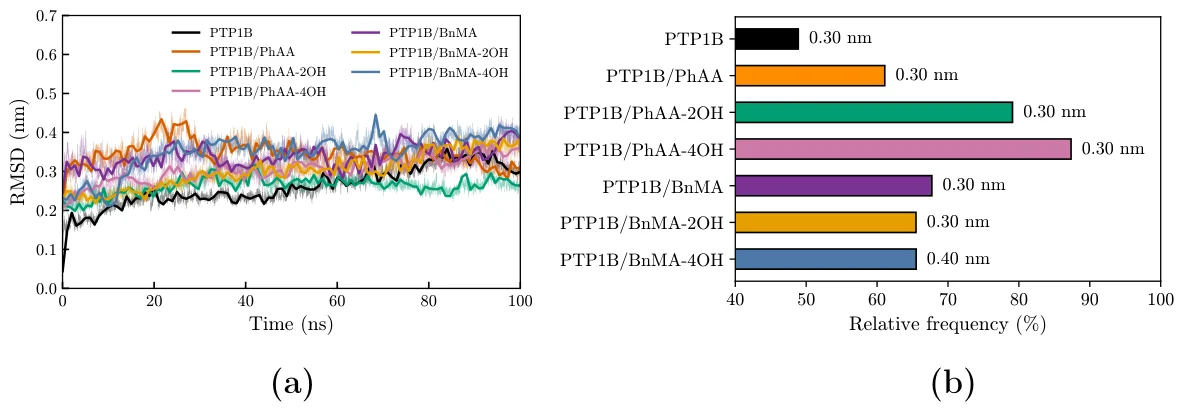
(**a**) C_α_ RMSD profiles of apo-PTP1B and the PTP1B–WSFD complexes during the 100 ns molecular dynamics simulations, and (**b**) relative frequency of the most frequently sampled RMSD value for each system.

**Figure 9:**
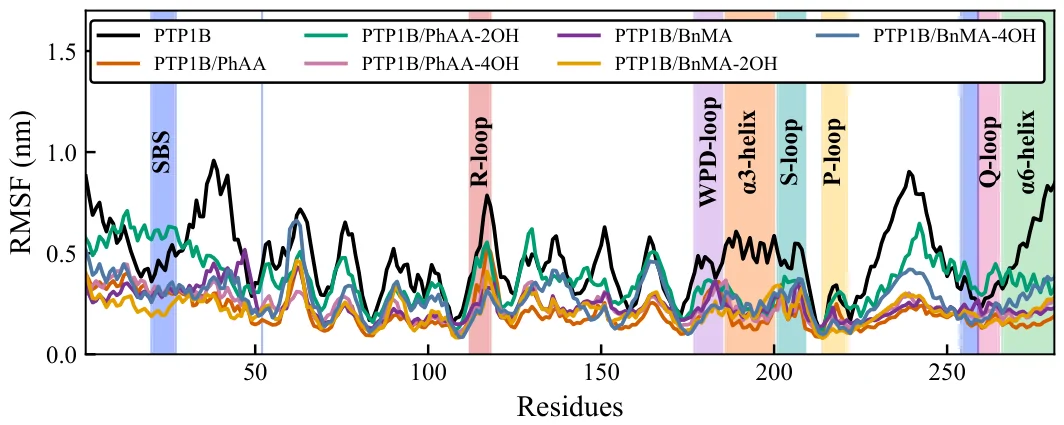
C_α_ RMSF profiles of residues 1–281 for apo-PTP1B and the studied PTP1B–WSFD complexes during the 100 ns molecular dynamics simulations. Relevant structural motifs are highlighted by colored regions within the graph.

**Figure 10:**
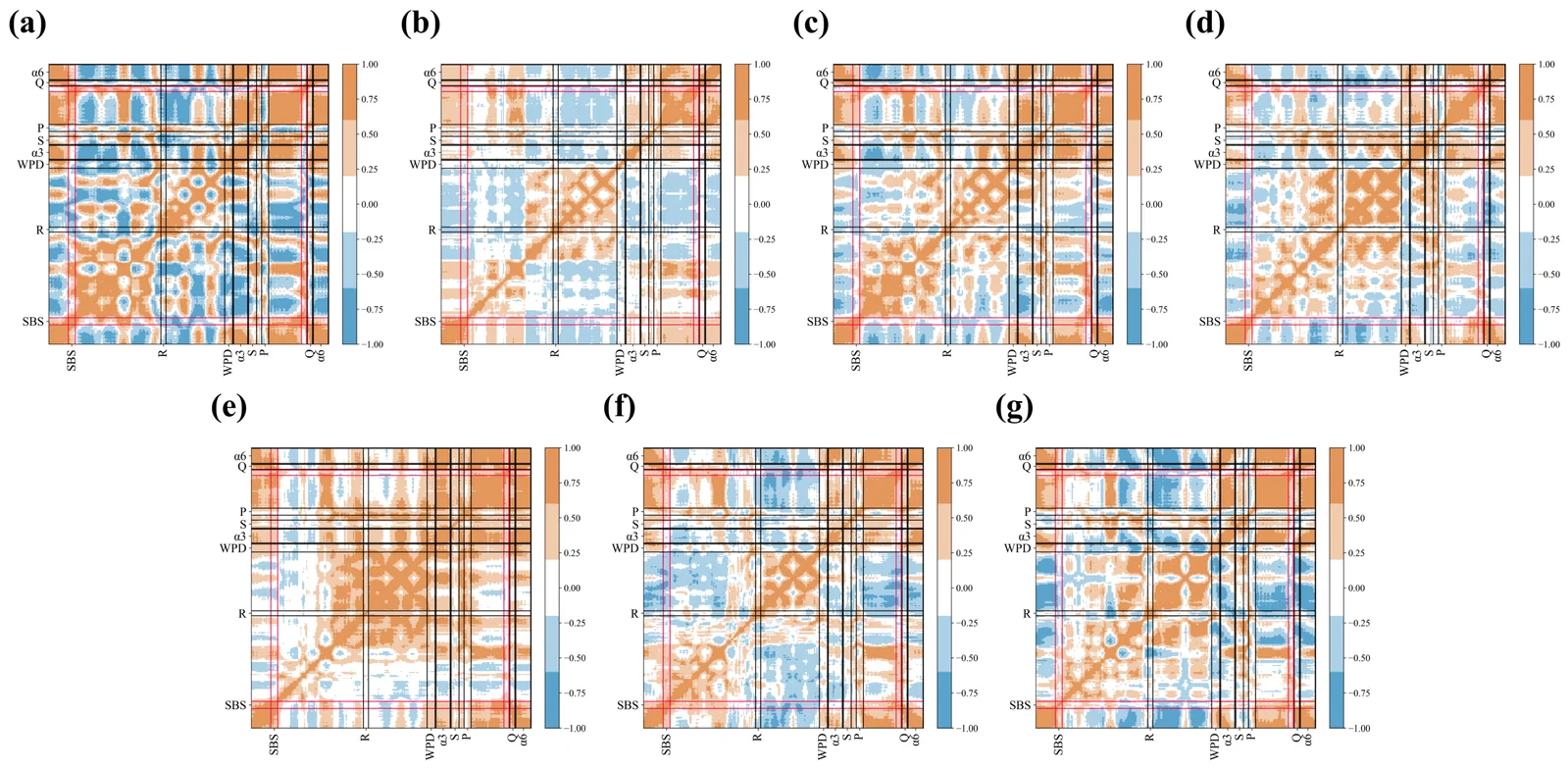
Dynamic cross-correlation matrices (DCCMs) of C_α_ motions for (**a**) apo-PTP1B, (**b**) PTP1B/PhAA, (**c**) PTP1B/PhAA-2OH, (**d**) PTP1B/PhAA-4OH, (**e**) PTP1B/BnMA, (**f**) PTP1B/ BnMA-2OH, and (**g**) PTP1B/BnMA-4OH. Positive and negative values indicate correlated and anticorrelated motions, respectively, according to the color scale. Relevant structural motifs are indicated along both axes. Colored guide lines delimit the residues associated with these structural motifs; red lines indicate motif regions composed of non-contiguous residues in the primary sequence.

**Figure 11:**
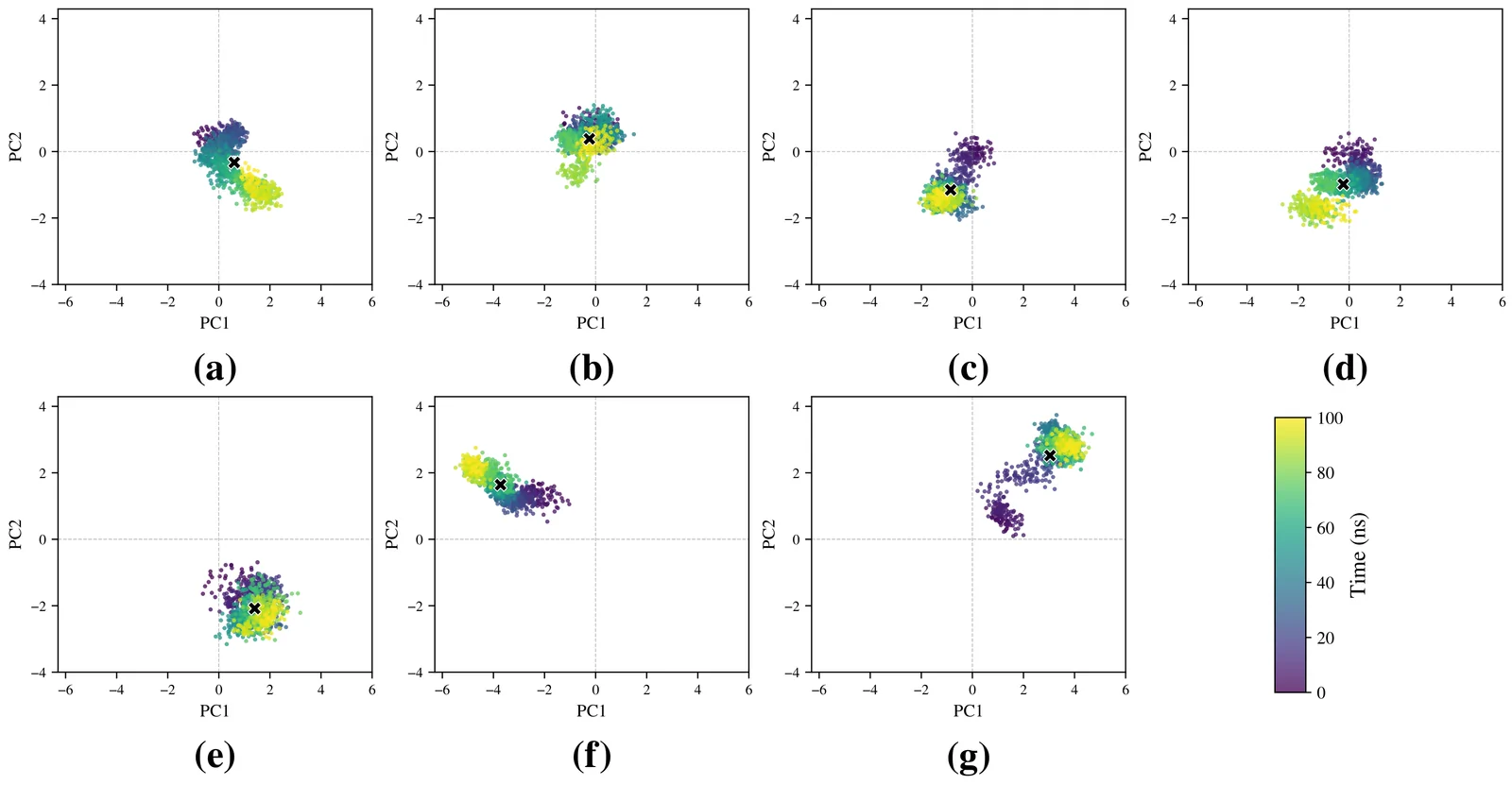
Projection of the C_α_ trajectories onto the common PC1–PC2 conformational space for (**a**) apo-PTP1B, (**b**) PTP1B/PhAA, (**c**) PTP1B/PhAA-2OH, (**d**) PTP1B/PhAA-4OH, (**e**) PTP1B/BnMA, (**f**) PTP1B/BnMA-2OH, and (**g**) PTP1B/BnMA-4OH. The color scale indicates the simulation time from 0 to 100 ns. The black cross in each panel indicates the centroid of the corresponding PC1–PC2 projection.

**Figure 12:**
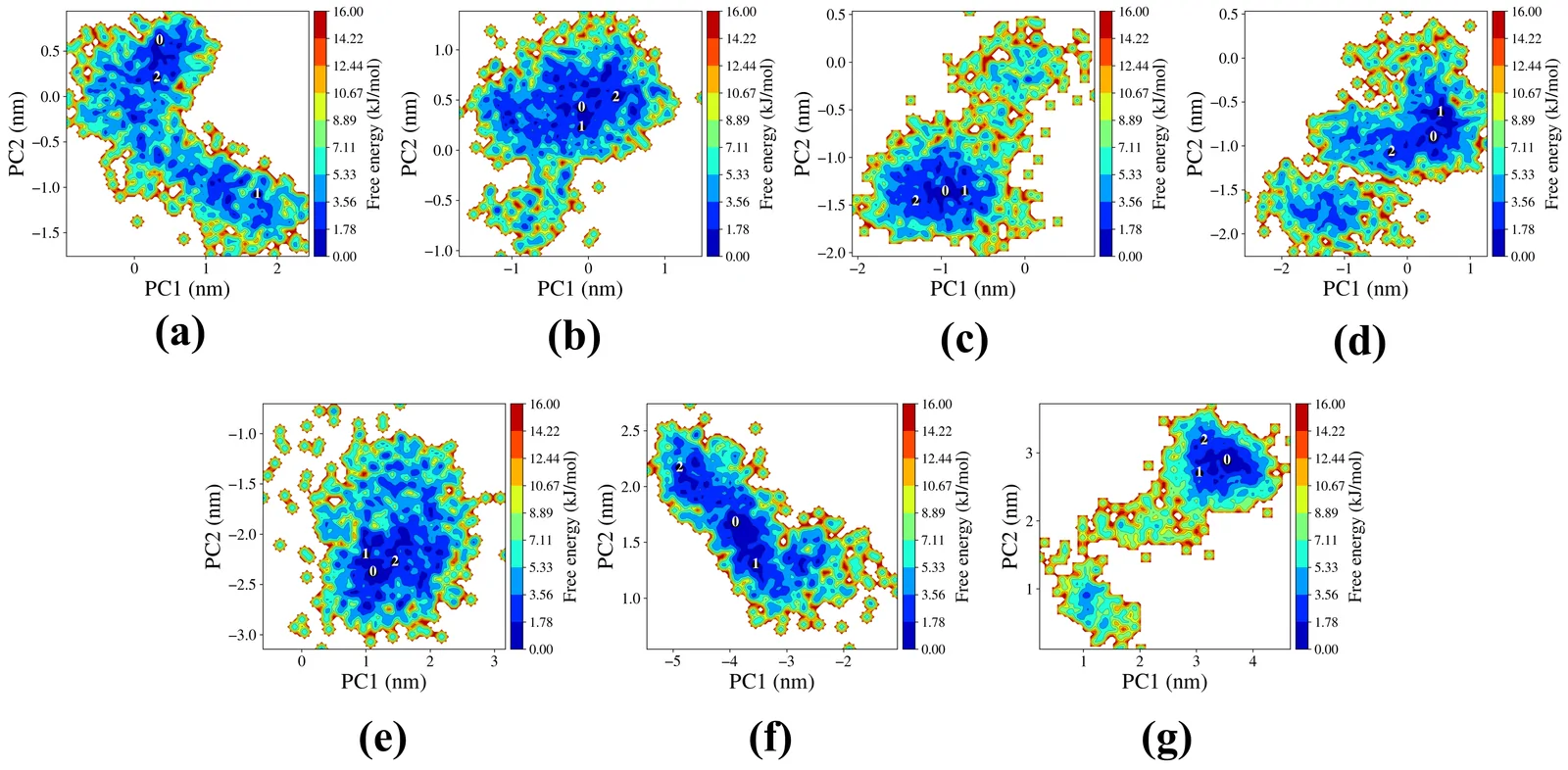
Free-energy landscapes (PCA-FEL) projected onto the common PC1–PC2 space for (**a**) apo-PTP1B, (**b**) PTP1B/PhAA, (**c**) PTP1B/PhAA-2OH, (**d**) PTP1B/PhAA-4OH, (**e**) PTP1B/BnMA, (**f**) PTP1B/BnMA-2OH, and (**g**) PTP1B/BnMA-4OH. The color scale represents the free energy in kJ/mol. Numbers 0, 1, and 2 indicate the locations in the PC1–PC2 space corresponding to the three lowest free-energy minima.

**Figure 13:**
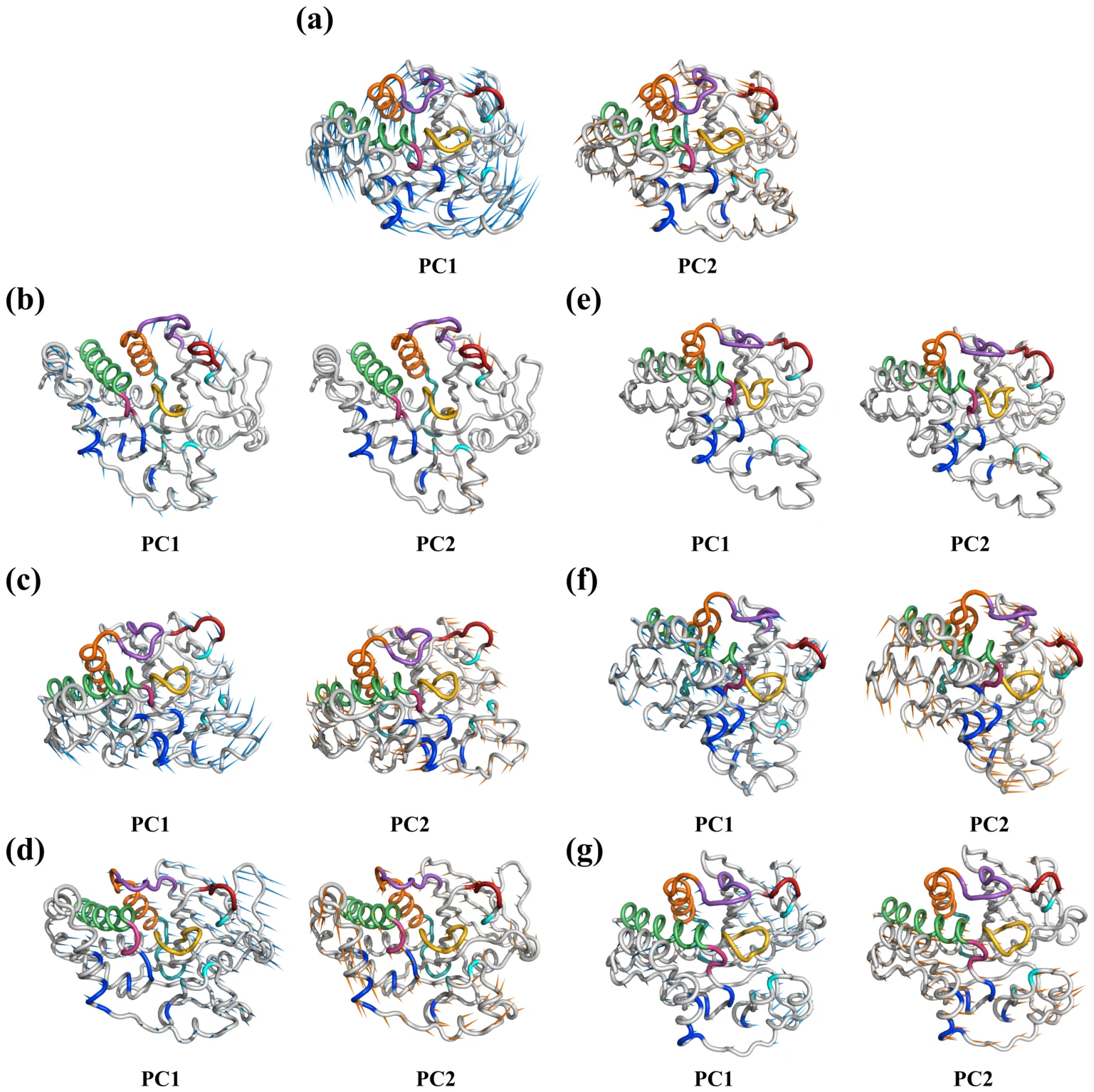
Principal component motions mapped onto the average structures of (**a**) apo-PTP1B, (**b**) PTP1B/PhAA, (**c**) PTP1B/PhAA-2OH, (**d**) PTP1B/PhAA-4OH, (**e**) PTP1B/BnMA, (**f**) PTP1B/BnMA-2OH, and (**g**) PTP1B/BnMA-4OH. The left and right columns show the displacement vectors associated with PC1 and PC2, respectively. Relevant structural motifs are highlighted as follows: WPD-loop (purple), P-loop (yellow), R-loop (red), α-helix 3 (orange), α-helix 6 (green), Q-loop (magenta), secondary binding site (dark blue), and substrate-recognition loop (cyan).

**Table 1:** Dipole moment (μ), reaction Gibbs free energy (ΔG), vertical ionization energy (*I*), and vertical electron affinity (*A*) for pristine C_60_ and the studied WSFDs.

System	μ (Debye)	ΔG (kcal/mol)	*I* (eV)	*A* (eV)
C_60_	0.00	–	7.61	2.46
PhAA	7.44	–	6.96	1.98
PhAA-2OH	6.43	−37.07 ^a^	6.81	1.95
PhAA-4OH	4.25	−43.45 ^b^	6.80	2.14
BnMA	4.52	–	7.00	2.04
BnMA-2OH	4.65	−37.18 ^a^	6.90	2.02
BnMA-4OH	5.45	−42.04 ^b^	6.78	2.13

^a^ ΔG for the reaction WSFD + H_2_O_2_ → WSFD(OH)_2_. ^b^ ΔG for the reaction WSFD(OH)_2_ + H_2_O_2_ → WSFD(OH)_4_.

**Table 2:** Average electrostatic potential (ESP) for the fullerene region, functionalization region, and whole molecule of the studied WSFDs.

Molecule	Fullerene Region (kcal/mol)	Functionalization Region (kcal/mol)	Overall ESP (kcal/mol)
PhAA	−0.14	−1.55	−0.97
PhAA-2OH	−0.15	−2.01	−1.22
PhAA-4OH	0.54	−0.53	−0.06
BnMA	1.15	−0.29	0.20
BnMA-2OH	1.29	−0.53	0.12
BnMA-4OH	0.23	−0.52	−0.24

**Table 3:** PTP1B residues involved in the interactions with PhAA, PhAA-2OH, and PhAA-4OH, together with their corresponding structural motifs or regions. Residues involved in hydrogen bonds are shown in **bold**, whereas hydrophobic contacts are shown in *italics*.

Ligand	Interacting Residues (Annotated)	Structural Motif/Region
PhAA	**Phe182**	WPD-loop
	*Thr178*, *Pro180*, *Asp181*	WPD-loop
	*Glu186*, *Ser187*, *Ser190*	α-helix 3
	*Arg112*, *Tyr152*	Adjacent/unclassified (pre-R region)
PhAA-2OH	**Gln127**, **Lys131**, **Asp137**	Adjacent to R-loop (substrate-recognition region)
	*Val113*	R-loop
	*Pro89*, *Leu119*, *Ala122*, *Gln123*, *Pro126*, *Met133*, *Ile134*, *Phe135*, *Glu136*	Adjacent to R-loop/N-terminal segment
PhAA-4OH	**Arg24**, *Gly259*	Secondary binding site
	*Asp48*, *Val49*	Substrate-recognition loop
	*Gly183*	WPD-loop
	**Arg221**, *Cys215*, *Ile219*, *Gly220*	Catalytic site
	**Gln262**, **Ala264**, **Asp265**, *Thr263*, **Gln266**	Q-loop/α-helix 6

**Table 4:** PTP1B residues involved in the interactions with BnMA, BnMA-2OH, and BnMA-4OH, together with their corresponding structural motifs or regions. Residues involved in hydrogen bonds are shown in **bold**, whereas hydrophobic contacts are shown in *italics*.

Ligand	Interacting Residues (Formatted by Interaction Type)	Structural Motif/Region
BnMA	*Tyr20*, **Arg24**, **Arg254**, *Gly259*	Secondary binding site
	**Trp179**, **Gly183**, *Phe182*	WPD-loop
	**Cys215**, *Ser216*, **Ala217**, *Ile219*, *Gly220*, **Arg221**	P-loop
	**Gln262**, *Thr263*, **Ala264**	Q-loop
	*Lys116*	R-loop
	*Tyr46*	Substrate-recognition loop
	**Ala17**, **Gln21**, *Asp48*	Adjacent/unclassified (near entry)
BnMA-2OH	*Tyr20*, **Arg24**, **Arg254**, *Gly259*	Secondary binding site
	**Trp179**, **Gly183**, *Phe182*	WPD-loop
	*Cys215*, *Ser216*, **Ala217**, **Arg221**, **Ile219**, *Gly220*	P-loop
	**Gln262**, *Thr263*, *Ala264*, **Asp265**	Q-loop
	*Lys116*	R-loop
	*Tyr46*	Substrate-recognition loop
	*Ala17*, *Gln123*, *Glu136*	Adjacent/unclassified (near active channel)
BnMA-4OH	*Tyr20*, **Arg24**, **Arg254**, *Gly259*	Secondary binding site
	**Gly183**, *Trp179*, *Phe182*	WPD-loop
	*Cys215*, *Ser216*, **Ala217**, *Ile219*, *Gly220*, **Arg221**	P-loop
	**Gln262**, **Gln266**	Q-loop/α-helix 6
	*Lys116*	R-loop
	*Tyr46*	Substrate-recognition loop
	**Gln21**, *Asp48*	Adjacent/unclassified (near entry)

**Table 5:** RMSD of the Cα atoms between the initial and average structures, together with the Phe182–Gly218 Cα distance for the initial ($d_{init}$) and average ($d_{ave}$) structures of apo-PTP1B and the studied PTP1B–WSFD complexes.

System	RMSD (Å)	$d_{\mathrm{init}}$ (Å)	$d_{\mathrm{ave}}$ (Å)
Apo	2.22	16.30	12.20
PhAA	2.61	16.50	20.50
PhAA-2OH	1.90	13.90	12.60
PhAA-4OH	1.97	16.80	18.00
BnMA	2.06	16.30	14.04
BnMA-2OH	1.99	18.10	19.00
BnMA-4OH	2.37	19.40	15.60

## Data Availability

The original/analyzed data needed to replicate these results cannot currently be distributed due to technical or time constraints. However, they can be made available by contacting the corresponding authors via email.

## Supplementary Materials

The following supporting information can be downloaded at https://www.mdpi.com/article/10.3390/molecules31193543/s1: Table S1: Calculated relative energies, dipole moments, and Gibbs free-energy changes (ΔG) for the structural isomers considered during the successive hydroxylation of PhAA and BnMA; Figure S1: Atom-resolved average electrostatic potential (ESP) for the studied WSFDs; Additional details and results of the redocking validation are provided in the Supplementary Materials [62,63,64]; Figure S2: Redocking of the co-crystallized inhibitor 989 in PTP1B and comparison between the crystallographic and redocked binding poses; Table S2: Docking scores and symmetry-corrected heavy-atom RMSD values for the 100 poses obtained from redocking of inhibitor 989; Figure S3: Clustering analysis of the molecular docking poses obtained for the PhAA-derived systems; Figure S4: Clustering analysis of the molecular docking poses obtained for the BnMA-derived systems; Figure S5: Backbone B-factor representation of apo-PTP1B and the PTP1B complexes with the PhAA-derived ligands; Figure S6: Backbone B-factor representation of apo-PTP1B and the PTP1B complexes with the BnMA-derived ligands; Figure S7: Time evolution of the solvent-accessible surface area (SASA) of apo-PTP1B and the PTP1B–ligand complexes; Figure S8: Time evolution of the radius of gyration ($R_{g}$) of apo-PTP1B and the PTP1B–ligand complexes; Figure S9: Time evolution of protein–ligand hydrogen bonds during the molecular dynamics simulations; Video S1: Molecular dynamics trajectory visualization of the PTP1B–PhAA complex; Video S2: Molecular dynamics trajectory visualization of the PTP1B–PhAA-2OH complex; Video S3: Molecular dynamics trajectory visualization of the PTP1B–PhAA-4OH complex; Video S4: Molecular dynamics trajectory visualization of the PTP1B–BnMA complex; Video S5: Molecular dynamics trajectory visualization of the PTP1B–BnMA-2OH complex; Video S6: Molecular dynamics trajectory visualization of the PTP1B–BnMA-4OH complex.
