# Supplementary material for: DFT, Molecular Docking, and Molecular Dynamics Analysis of Electronic Structure, Reactivity, and PTP1B Binding of Phenylacetic and Benzylmalonic Acid-Functionalized C60 Derivatives
Source: Molecules. 2026 Oct 4;31(19):3543. doi: 10.3390/molecules31193543 (PMC13667046; doi:10.3390/molecules31193543)
Supplement: Supplementary file 1 [file molecules-31-03543-s001.zip › SI_ptp1b_full_deriv.pdf]

Supplementary Material for:

DFT, Molecular Docking, and Molecular Dynamics Analysis of Electronic Structure, Reactivity, and PTP1B Binding of Phenylacetic and Benzylmalonic Acid-Functionalized C_{60} Derivatives

Verónica Rodríguez-Celestino [a], Zuriel Natanael Cisneros-García[a,*], Jaime Gustavo Rodríguez-Zavala[a,*], Carlos Iván Méndez-Barrientos[a], and José Guadalupe Facio-Muñoz

[a] Universidad de Guadalajara, Centro Universitario de los Lagos, Departamento de Ciencias Exactas y Tecnología. Av. Enrique Díaz de León 1144, Lagos de Moreno, Jalisco, 47460, Mexico.

[*] E-mail address: zuriel.cisneros8072@academicos.udg.mx (Cisneros-García Z. N.); jaime.rzavala@academicos.udg.mx (Rodríguez-Zavala J. G.)

S1 Electronic structure

S1.1 Hydroxylation of PhAA and BnMA

Table S1: Calculated relative energies, dipole moments, and Gibbs free-energy changes (ΔG) for the structural isomers considered during the successive hydroxylation of PhAA and BnMA. The atom pairs indicate the fullerene carbon atoms considered for hydroxyl addition and their relative positions on the C_{60} cage (para, [6,6], or [5,6]). Relative energies are reported with respect to the lowest-energy isomer within each set, which is assigned a value of 0.00 kcal mol^{-1}. For the dihydroxylated derivatives, ΔG corresponds to the reaction C60–X + H_2O2 → C60(OH)2–X, whereas for the tetrahydroxylated derivatives it corresponds to C60$(OH)_2$–X + H_2O2 → C60$(OH)_4$–X. Relative energies and ΔG values are expressed in kcal mol^{-1} and dipole moments in D.

**Isomer**	**Atom pair**	**Relative energy (kcal mol^{-1})**	**Dipole moment (D)**	**ΔG (kcal mol^{-1})**
PhAA-2OH Iso 1	C25–C17 (para)	6.16	5.94	−30.51
PhAA-2OH Iso 2	C25–C52 (para)	6.55	6.63	−29.93
PhAA-2OH Iso 3	C25–C24 (6,6)	0.00	6.43	**–37.07**
PhAA-2OH Iso 4	C25–C26 (5,6)	13.66	7.99	−23.74
PhAA-4OH Iso 1	C20–C10 (para)	42.93	7.80	−14.30
PhAA-4OH Iso 2	C20–C19 (para)	12.66	5.02	−36.28
PhAA-4OH Iso 3	C20–C8 (6,6)	0.00	4.25	**–43.45**
PhAA-4OH Iso 4	C20–C21 (5,6)	22.99	6.77	−26.90
BnMA-2OH Iso 1	C24–C51 (para)	7.18	4.56	-31.87
BnMA-2OH Iso 2	C24–C16 (para)	8.50	4.67	-29.38
BnMA-2OH Iso 3	C24–C25 (6,6)	0.00	4.65	**-37.18**
BnMA-2OH Iso 4	C24–C23 (5,6)	38.08	6.43	-6.42
BnMA-4OH Iso 1	C37–C40 (para)	26.74	12.10	-18.28
BnMA-4OH Iso 2	C37–C27 (para)	7.41	4.13	-35.00
BnMA-4OH Iso 3	C37–C38 (6,6)	0.00	5.45	**-42.04**
BnMA-4OH Iso 4	C37–C36 (5,6)	39.64	3.13	-13.22

S1.2 Average ESP statistics

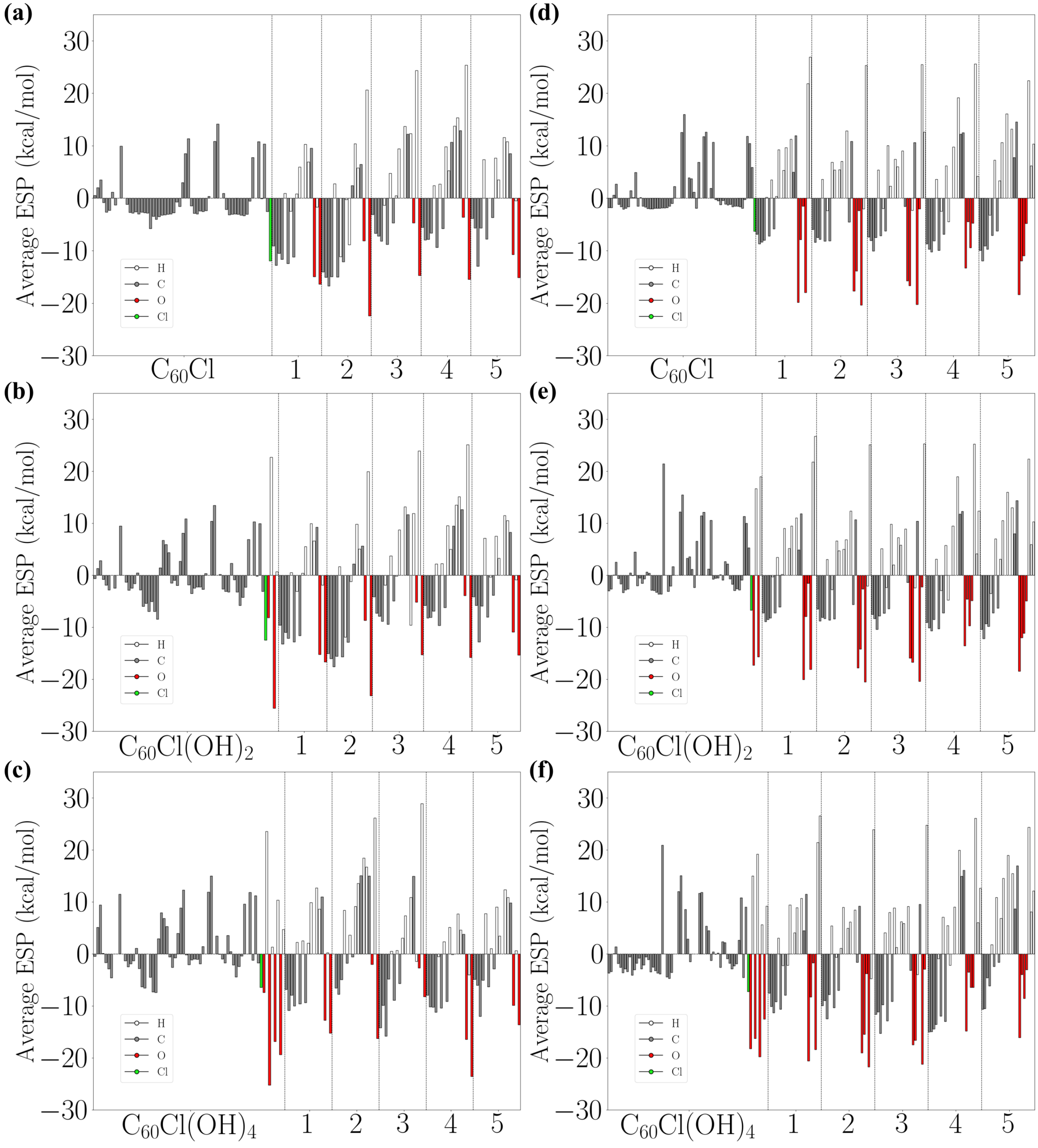

Figure S1: Atom-resolved average electrostatic potential (ESP) for (a) PhAA, (b) PhAA-2OH, (c) PhAA-4OH, (d) BnMA, (e) BnMA-2OH, and (f) BnMA-4OH. In each panel, the atoms are separated into the fullerene region, comprising the C_{60} cage, the covalently bound Cl atom, and the hydroxyl groups when present, and the functionalization region, comprising the five phenylacetic- or benzylmalonic-acid-derived substituents, numbered from 1 to 5. Bars correspond to the atom-averaged ESP values for H, C, O, and Cl atoms, expressed in kcal mol^{-1}; positive and negative values indicate the local electrostatic character of the corresponding atomic regions.

S2 Molecular Docking

S2.1 Redocking

Redocking of the co-crystallized inhibitor 989 was performed using five independent AutoDock Vina calculations, with 20 poses retained from each run, yielding a total of 100 poses. The agreement between the crystallographic and redocked poses was evaluated using the symmetry-corrected heavy-atom RMSD, which accounts for equivalent atomic arrangements that may otherwise artificially increase the calculated RMSD [1]. The resulting docking scores and symmetry-corrected heavy-atom RMSD values are reported in Table S2. Although the generated poses span a relatively broad RMSD distribution, the crystallographic binding mode was repeatedly recovered during the docking calculations. The lowest symmetry-corrected RMSD was 1.19 Å (Figure S2), and five poses showed RMSD values below 1.5 Å, with at least one pose identified in each independent run. RMSD values below approximately 2 Å are commonly considered indicative of successful reproduction of a crystallographic binding pose [2]. However, ligand size and conformational flexibility can affect the magnitude and interpretation of RMSD and can increase the difficulty of reproducing the experimental pose during flexible-ligand docking [3]. Therefore, the consistent recovery of poses with RMSD below 1.5 Å indicates that the sampling protocol is capable of reproducing the experimentally observed binding geometry.

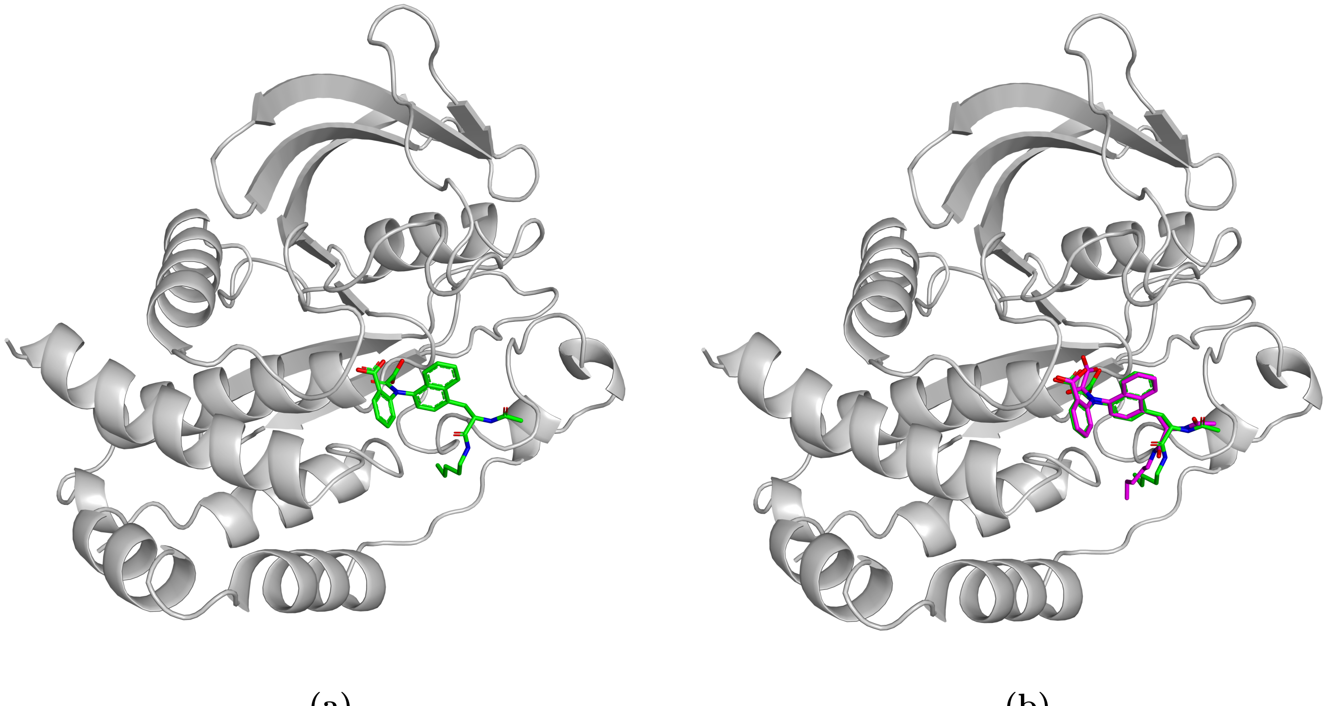

(a) (b)

Figure S2: Redocking of the co-crystallized inhibitor 989 in PTP1B (PDB ID: 1NL9). (a) Crystallographic binding pose of inhibitor 989 within the PTP1B catalytic region. (b) Superposition of the crystallographic pose and the best pose recovered by AutoDock Vina redocking. The minimum symmetry-corrected RMSD between the redocked and crystallographic poses was 1.19 Å. PTP1B is shown in gray cartoon representation.

Table S2: Docking scores and symmetry-corrected heavy-atom RMSD values for the 100 poses obtained from redocking of the co-crystallized inhibitor 989 in PTP1B (PDB ID: 1NL9). Poses are grouped by independent docking run and ordered by pose number. Docking scores are given in kcal mol^{-1} and RMSD values in Å. RMSD values below 2.0 Å are shown in bold.

No.	Run	Pose	Score	RMSD	No.	Run	Pose	Score	RMSD
1	1	1	-7.4	6.332	51	3	11	-6.8	7.578
2	1	2	-7.3	6.297	52	3	12	-6.8	4.469
3	1	3	-7.2	5.710	53	3	13	-6.8	6.014
4	1	4	-7.2	6.398	54	3	14	-6.7	5.137
5	1	5	-7.1	3.862	55	3	15	-6.7	5.799
6	1	6	-7.1	**1.289**	56	3	16	-6.7	4.943
7	1	7	-7.0	2.172	57	3	17	-6.6	8.803
8	1	8	-7.0	8.374	58	3	18	-6.6	4.051
9	1	9	-7.0	4.400	59	3	19	-6.6	8.085
10	1	10	-6.9	2.838	60	3	20	-6.6	4.075
11	1	11	-6.9	4.272	61	4	1	-7.4	6.332
12	1	12	-6.8	8.364	62	4	2	-7.2	5.486
13	1	13	-6.8	7.543	63	4	3	-7.1	**1.203**
14	1	14	-6.8	5.041	64	4	4	-7.1	6.385
15	1	15	-6.8	4.136	65	4	5	-7.0	3.844
16	1	16	-6.8	4.771	66	4	6	-7.0	3.015
17	1	17	-6.7	8.768	67	4	7	-6.9	4.988
18	1	18	-6.7	5.808	68	4	8	-6.9	2.122
19	1	19	-6.7	8.123	69	4	9	-6.9	6.334
20	1	20	-6.6	5.806	70	4	10	-6.8	5.196
21	2	1	-7.5	6.451	71	4	11	-6.8	6.207
22	2	2	-7.4	6.332	72	4	12	-6.8	8.273
23	2	3	-7.3	6.506	73	4	13	-6.8	6.012
24	2	4	-7.3	**1.186**	74	4	14	-6.8	4.250
25	2	5	-7.3	5.699	75	4	15	-6.7	4.126
26	2	6	-7.2	3.883	76	4	16	-6.7	5.583
27	2	7	-7.2	2.167	77	4	17	-6.7	4.315
28	2	8	-7.2	4.991	78	4	18	-6.7	6.134
29	2	9	-7.1	8.255	79	4	19	-6.7	8.843
30	2	10	-7.1	4.373	80	4	20	-6.6	5.258
31	2	11	-7.1	6.333	81	5	1	-7.4	5.755
32	2	12	-7.0	4.371	82	5	2	-7.4	6.221
33	2	13	-6.9	5.578	83	5	3	-7.4	6.500
34	2	14	-6.9	6.337	84	5	4	-7.4	6.388
35	2	15	-6.9	8.776	85	5	5	-7.3	**1.235**
36	2	16	-6.9	5.800	86	5	6	-7.3	2.152
37	2	17	-6.9	5.969	87	5	7	-7.2	3.884
38	2	18	-6.9	6.155	88	5	8	-7.2	4.973
39	2	19	-6.9	8.131	89	5	9	-7.2	8.378
40	2	20	-6.8	4.211	90	5	10	-7.1	4.124
41	3	1	-7.4	6.336	91	5	11	-7.1	8.344
42	3	2	-7.2	5.473	92	5	12	-7.1	5.651
43	3	3	-7.2	6.550	93	5	13	-7.0	4.152
44	3	4	-7.1	**1.212**	94	5	14	-7.0	7.659
45	3	5	-7.1	3.748	95	5	15	-7.0	7.534
46	3	6	-7.0	4.992	96	5	16	-7.0	5.762
47	3	7	-7.0	2.103	97	5	17	-6.9	8.131
48	3	8	-6.9	8.405	98	5	18	-6.9	3.757
49	3	9	-6.9	2.815	99	5	19	-6.9	8.817
50	3	10	-6.9	8.284	100	5	20	-6.8	5.810

S2.2 Clustering analysis

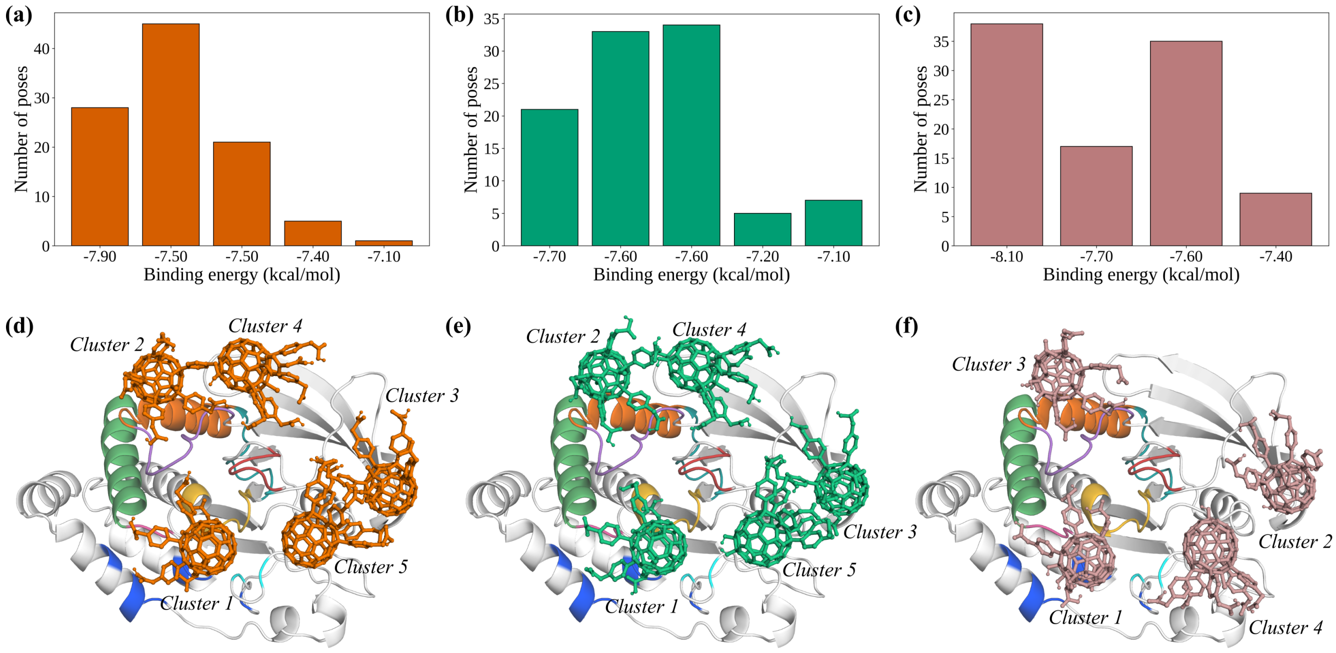

Figure S3: Clustering analysis of the molecular docking poses obtained for the PhAA-derived systems: (a,d) PhAA, (b,e) PhAA-2OH, and (c,f) PhAA-4OH. Panels (a–c) show the number of poses assigned to each cluster as a function of the minimum binding energy identified for that cluster, expressed in kcal mol^{-1}. Panels (d–f) show the spatial distribution of the identified clusters over the PTP1B catalytic domain. For each ligand, 100 docking poses generated from independent AutoDock Vina calculations were grouped by hierarchical clustering using an RMSD cutoff of 8 Å.

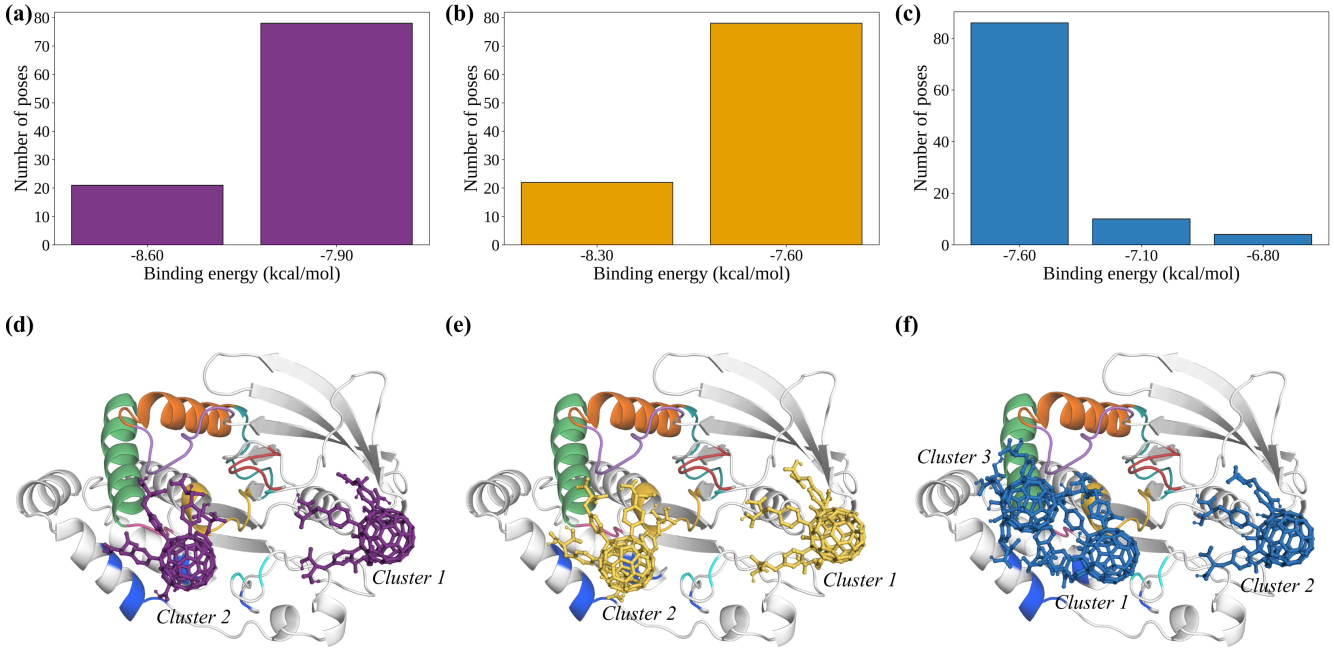

Figure S4: Clustering analysis of the molecular docking poses obtained for the BnMA-derived systems: (a,d) BnMA, (b,e) BnMA-2OH, and (c,f) BnMA-4OH. Panels (a–c) show the number of poses assigned to each cluster as a function of the minimum binding energy identified for that cluster, expressed in kcal mol^{-1}. Panels (d–f) show the spatial distribution of the identified clusters over the PTP1B catalytic domain. For each ligand, 100 docking poses generated from independent AutoDock Vina calculations were grouped by hierarchical clustering using an RMSD cutoff of 8 Å.

S3 Molecular dynamics

S3.1 B-Factor

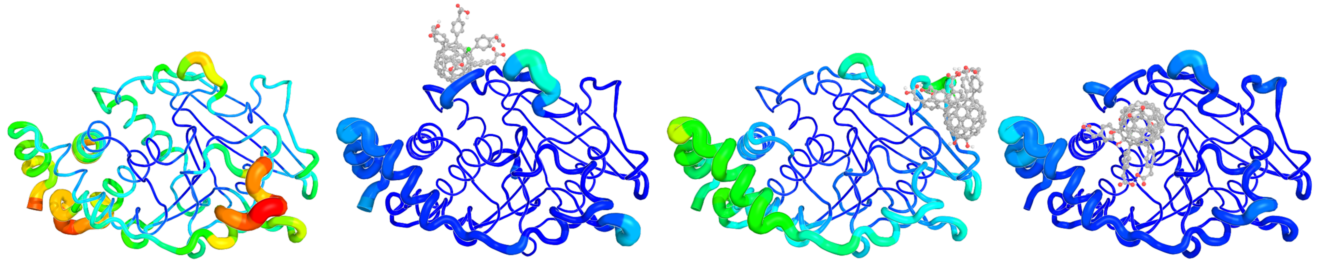

Figure S5: Backbone B-factor representation of apo-PTP1B and the PTP1B complexes with the PhAA-derived ligands over the 100 ns molecular dynamics simulations. Structures are shown from left to right for apo-PTP1B, PTP1B/PhAA, PTP1B/PhAA-2OH, and PTP1B/PhAA-4OH. The representation is restricted to residues 1–281 of PTP1B. The color scale is referenced to the mobility of the apo protein, whereas the backbone thickness highlights regions exhibiting larger fluctuations within each system. Ligands are displayed together with the protein structure to indicate their binding location.

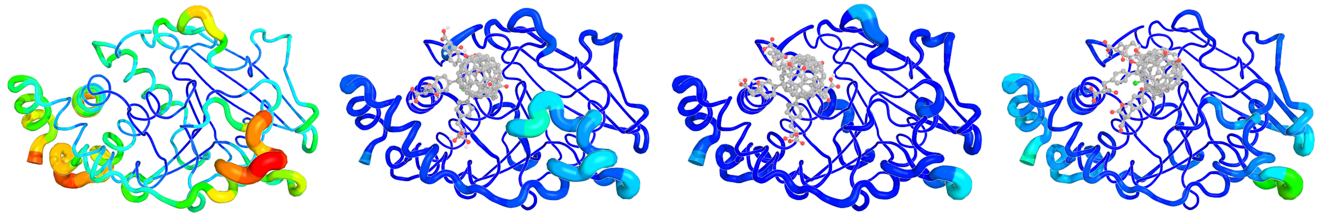

Figure S6: Backbone B-factor representation of apo-PTP1B and the PTP1B complexes with the BnMA-derived ligands over the 100 ns molecular dynamics simulations. Structures are shown from left to right for apo-PTP1B, PTP1B/BnMA, PTP1B/BnMA-2OH, and PTP1B/BnMA-4OH. The representation is restricted to residues 1–281 of PTP1B. The color scale is referenced to the mobility of the apo protein, whereas the backbone thickness highlights regions exhibiting larger fluctuations within each system. Ligands are displayed together with the protein structure to indicate their binding location.

S3.2 SASA, Rg and Hydrogen-bonds

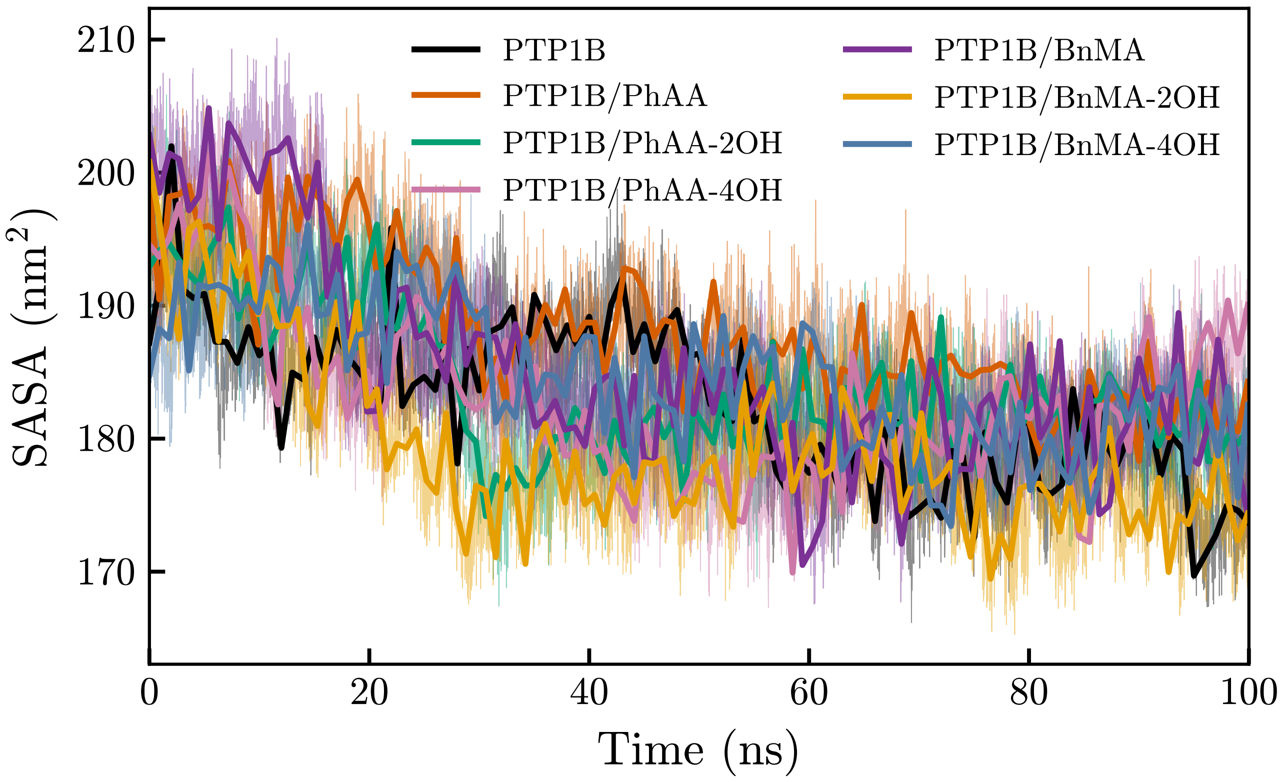

Figure S7: Time evolution of the solvent-accessible surface area (SASA) of PTP1B during the 100 ns molecular dynamics simulations of the apo-PTP1B and the complexes formed with PhAA, PhAA-2OH, PhAA-4OH, BnMA, BnMA-2OH, and BnMA-4OH. SASA values are reported in nm^2 and describe the variation in the solvent-exposed surface of the protein throughout each trajectory.

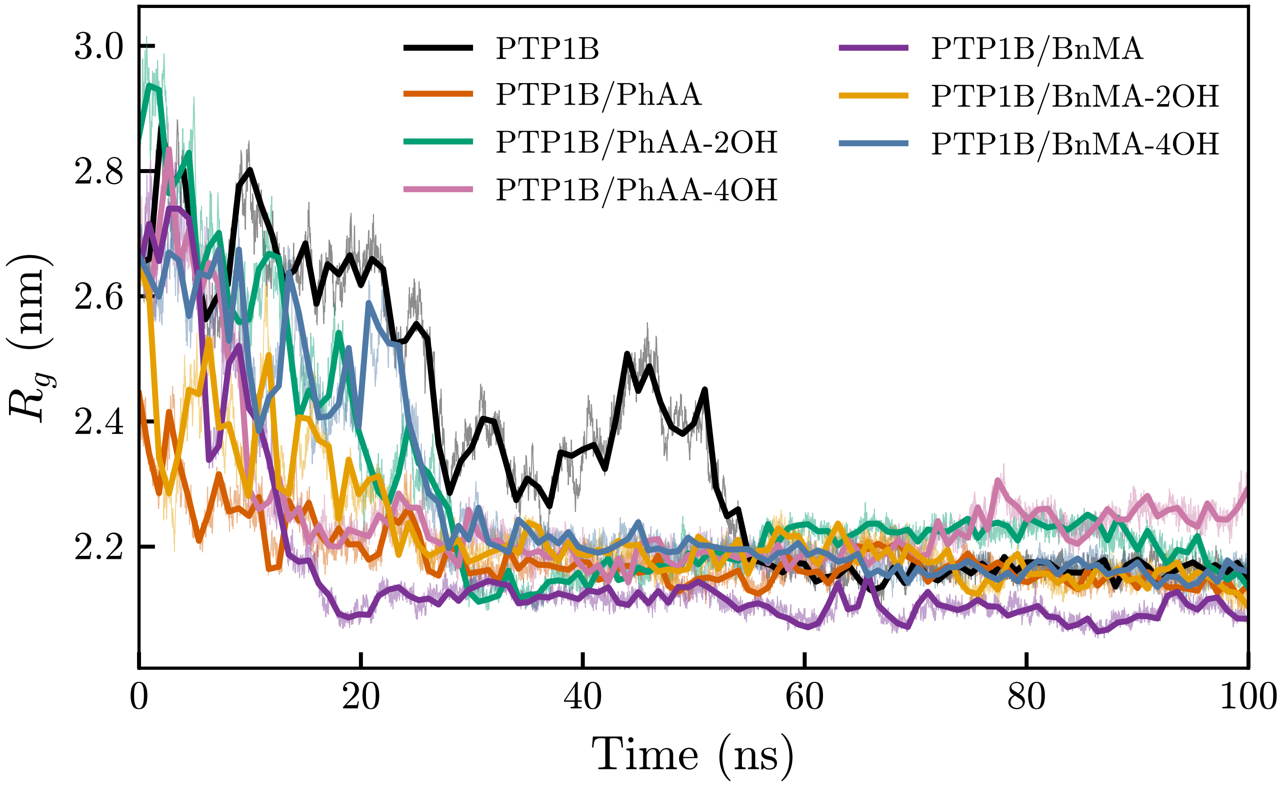

Figure S8: Time evolution of the radius of gyration (R_g) of PTP1B during the 100 ns molecular dynamics simulations of the apo protein (PTP1B) and the complexes formed with PhAA, PhAA-2OH, PhAA-4OH, BnMA, BnMA-2OH, and BnMA-4OH. R_g values are reported in nm and describe changes in the overall compactness of the protein throughout each trajectory.

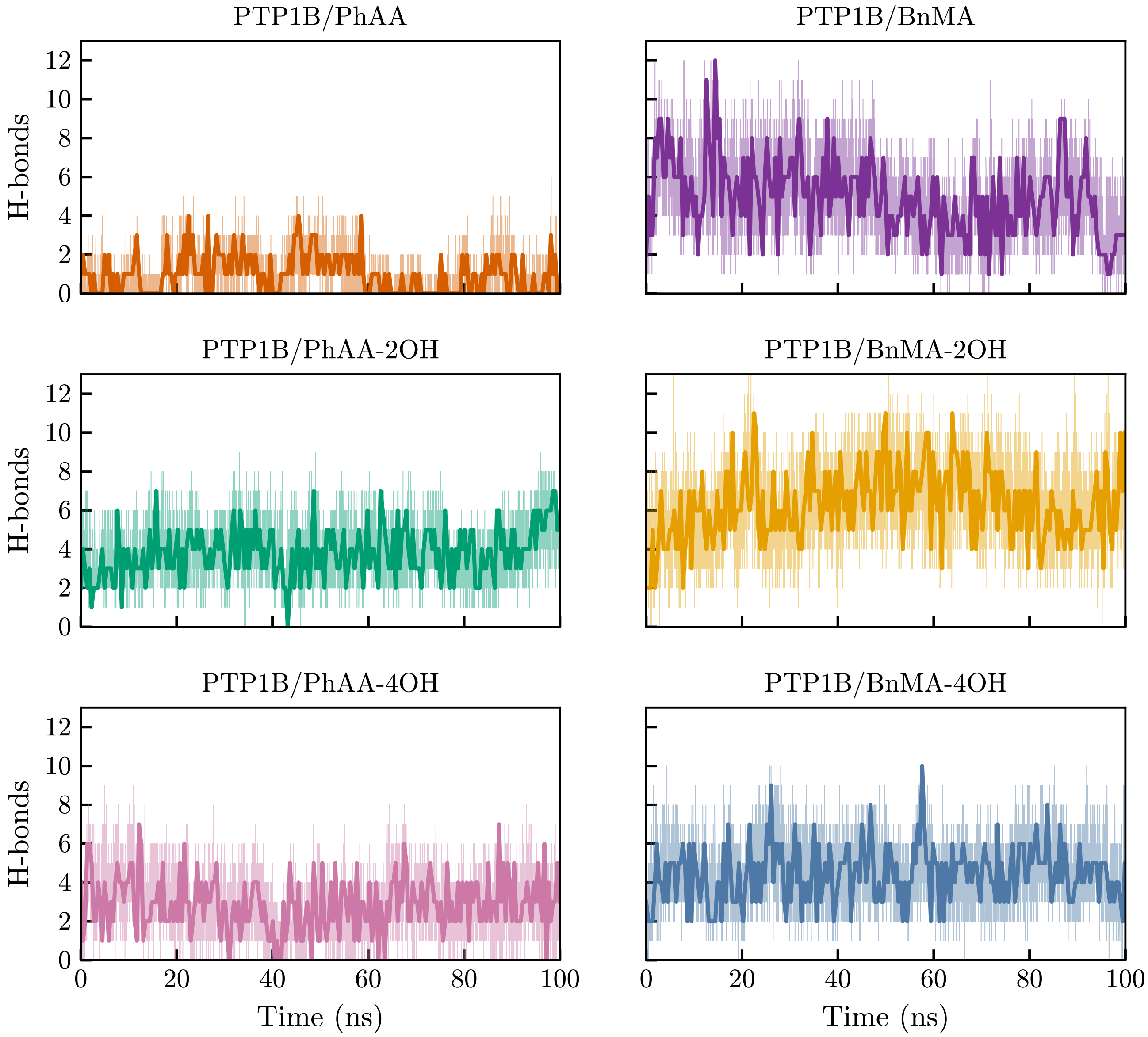

Figure S9: Time evolution of the number of hydrogen bonds formed between PTP1B and each fullerene derivative during the 100 ns molecular dynamics simulations. The panels correspond to the PTP1B/PhAA, PTP1B/PhAA-2OH, PTP1B/PhAA-4OH, PTP1B/BnMA, PTP1B/BnMA-2OH, and PTP1B/BnMA-4OH complexes. The profiles illustrate the formation and fluctuation of protein–ligand hydrogen-bond interactions throughout each trajectory.

S4 References

References

[1] Bell, E.W.; Zhang, Y. DockRMSD: An Open-Source Tool for Atom Mapping and RMSD Calculation of Symmetric Molecules through Graph Isomorphism. *J. Cheminform.* **2019**, *11*, 40. https://doi.org/10.1186/s13321-019-0362-7.

[2] Damm-Ganamet, K.L.; Smith, R.D.; Dunbar, J.B., Jr.; Stuckey, J.A.; Carlson, H.A. CSAR Benchmark Exercise 2011–2012: Evaluation of Results from Docking and Relative Ranking of Blinded Congeneric Series. *J. Chem. Inf. Model.* **2013**, *53*, 1853–1870. https://doi.org/10.1021/ci400025f.

[3] Boittier, E.D.; Tang, Y.Y.; Buckley, M.E.; Schuurs, Z.P.; Richard, D.J.; Gandhi, N.S. Assessing Molecular Docking Tools to Guide Targeted Drug Discovery of CD38 Inhibitors. *Int. J. Mol. Sci.* **2020**, *21*, 5183. https://doi.org/10.3390/ijms21155183.
